# Bufalin inhibits hepatocellular carcinoma progression by blocking EGFR-mediated RAS-RAF-MEK-ERK pathway activation

**DOI:** 10.1186/s13046-025-03531-3

**Published:** 2025-08-29

**Authors:** Jingwen Liu, Jia Jiang, Ju Huang, Zhi-E Fang, Lexi Liu, Yong Liu, Weiqi Nian, Jianyuan Tang, Zhilei Wang

**Affiliations:** 1 Hospital of Chengdu University of Traditional Chinese Medicine, Chengdu, 610075 China; 2 Department of Oncology, Chongqing Traditional Chinese Medicine Hospital, Chongqing, 400021 China; 3 TCM Prevention and Treatment of Metabolic and Chronic Diseases Key Laboratory of Sichuan Province, Hospital of Chengdu University of Traditional Chinese Medicine, Chengdu, 610075 China

**Keywords:** Hepatocellular carcinoma, Cinobufacini preparations, Bufalin, RAS/RAF/MEK/ERK, EGFR

## Abstract

**Background:**

Hepatocellular carcinoma (HCC) remains one of the most challenging malignancies with persistently dismal long-term survival outcomes despite multidisciplinary advances in diagnostic and therapeutic strategies. Cinobufacini preparations have garnered increasing attention as adjunctive therapeutic agents in integrated management strategies for HCC. Bufalin (BF), the active ingredient in Cinobufacini, has garnered substantial attention due to its potent antitumor effects. However, the precise molecular mechanisms underlying its antitumor actions remain incompletely characterized.

**Methods:**

A clinical retrospective cohort analysis was conducted to establish the definitive clinical benefit of Cinobufacini in improving treatment outcomes among HCC patients. Building upon these clinical insights, a multi-dimensional approach was implemented to elucidate the anti-HCC molecular mechanisms mediated by the bioactive component BF of Cinobufacini.

**Results:**

Western medical treatment combined with Cinobufacini shows an improving trend in the overall survival (OS) and progression free survival (PFS) of HCC patients. Moreover, our exploratory analysis suggests a potential dose-response relationship where longer cumulative exposure to Cinobufacini appears to be associated with improved clinical outcomes. In vitro experiments demonstrated that BF significantly inhibited cell viability and proliferation, and induced apoptosis in HepG2 and HCCLM3. Network pharmacology analysis identified 20 core targets, and molecular docking revealed high-affinity binding between BF and key proteins, including EGFR, GRB2, SRC, and MAPK1. HCC tissue microarrays confirmed the overexpression of EGFR and GRB2 in HCC tissues. Further mechanistic investigations revealed that BF suppressed the EGFR-mediated RAS/RAF/MEK/ERK pathway activation in HepG2 and HCCLM3. BF intervention significantly reduced tumor volumes in C57BL/6 mouse subcutaneous HCC xenograft and BALB/c Nude mouse orthotopic HCC xenograft models. Moreover, BF inhibited the phosphorylation levels of EGFR, RAF, MEK, and ERK in tumor tissues, further corroborating its inhibitory effects on the RAS/RAF/MEK/ERK signaling pathway.

**Conclusions:**

Our observational data suggest a potential association between Cinobufacini use and favorable trends in OS and PFS among HCC patients. BF exerts its antitumor effects against HCC by interfering with the EGFR-mediated RAS/RAF/MEK/ERK signaling pathway. These findings not only elucidate the molecular mechanisms underlying the antitumor actions of BF but also highlight the potential of Cinobufacini preparations as a valuable therapeutic option for HCC.

**Supplementary Information:**

The online version contains supplementary material available at 10.1186/s13046-025-03531-3.

## Background

Primary Liver Cancer (PLC) is a malignant tumor with high incidence and mortality rates worldwide, posing a severe threat to human life and health. The complex pathogenesis and diverse pathological subtypes of PLC pose substantial challenges for clinical diagnosis and treatment [1, 2]. Hepatocellular carcinoma (HCC), the most predominant pathological subtype of PLC, accounting for approximately 75% − 85% of all cases, is the most common and lethal type within PLC [3]. The occurrence of HCC is closely associated with chronic liver diseases, and common risk factors include infection with hepatitis B virus (HBV), hepatitis C virus (HCV), alcoholic liver disease (ALD), non-alcoholic fatty liver disease (NAFLD), as well as aflatoxin exposure [4–8]. Although surgical resection, liver transplantation, and local ablation therapies provide a curative intent for early-stage HCC, most patients present with advanced disease at diagnosis, having already lost the opportunity for surgical intervention. Advanced-stage HCC primarily relies on targeted therapies (such as sorafenib and lenvatinib) and immunotherapies (such as PD-1/PD-L1 inhibitors) [9–11]. However, due to the limited efficacy of current therapeutic strategies and the propensity for drug resistance, the median survival time of patients with advanced HCC remains low, and the 5-year survival rate is suboptimal. Therefore, exploring more effective methods for early diagnosis and treatment strategies is currently a focal point of research.

Venenum Bufonis is a traditional Chinese medicine derived from the white secretions processed and dried from the postauricular and cutaneous glands of toads in the *Bufo bufo gargarizans* Cantor or *Bufo melanostictus* Schneider. It serves as a crucial component in various traditional Chinese medicine formulas. Cinobufacini Capsules and Cinobufacini Tablets, independently developed and manufactured in China, are antitumor preparations extracted from Venenum Bufonis. They are utilized in the treatment of various malignant tumors, including liver cancer, gastric cancer, and colorectal cancer [12–14]. Bufalin (BF), as the core active ingredient in Cinobufacini preparations, exhibits antitumor effects against a variety of solid tumors and hematological malignancies, such as ovarian cancer, HCC, breast cancer, and prostate cancer [15–19], but the underlying mechanisms by which it exerts its antitumor effects against HCC still require in-depth exploration.

To address the issues, we propose to establish a comprehensive research framework integrating “clinical efficacy validation and molecular mechanism elucidation”. This framework aims to reveal the clinical value of Cinobufacini preparations comprehensively and deeply in enhancing treatment outcomes for HCC patients. Meanwhile, we will employ a multi-dimensional approach encompassing network pharmacology, molecular docking, HCC tissue microarrays, in vitro cell-based experimental validation, and in vivo animal experimental evaluation. Through these methods, we will conduct an in-depth analysis of the therapeutic efficacy and target mechanisms of BF in combating HCC. By utilizing this comprehensive research framework, we intend to unveil the clinical value of Cinobufacini in the treatment of HCC and delve into the action mechanisms of BF against HCC. This will provide a robust theoretical foundation and practical guidance for overcoming the current challenges in HCC treatment.

## Methods

### Cell culture

Human HCC cell lines HepG2 and HCCLM3 were obtained from the Shanghai Institute of Biochemistry and Cell Biology. Mouse Hepa1-6 and human HepG2-Luc (an HCC cell line with luciferase expression) were provided by Wuhan Pricella Biotechnology Co., Ltd. HepG2 cells and HepG2-Luc cells were grown in Minimum Essential Medium (MEM, Gibco, 11095080). HCCLM3 and Hepa1-6 cells were grown in Dulbecco’s Modified Eagle Medium (DMEM, Gibco, 11965092). The culture medium was supplemented with 10% fetal bovine serum (FBS, Gibco, 10100), 1% penicillin/streptomycin solution (Solarbio, P1400). Cell cultures were maintained in a controlled environment at 37 °C with 5% (v/v) CO₂.

### Retrospective cohort study

Retrospective cohort study was approved by the Ethics Committee of the Hospital of Chengdu University of Traditional Chinese Medicine (Approval No.: 2024KL-123, Clinical trial number: not applicable). We collected clinical data from inpatients who were first diagnosed with HCC at the hospital of Chengdu University of Traditional Chinese Medicine between January 2018 and December 2023. The data were rigorously screened according to the diagnostic criteria, inclusion criteria, and exclusion criteria outlined in the “Guidelines for the Diagnosis and Treatment of Primary Liver Cancer (2024 Edition)”. Through propensity score matching (PSM), we ultimately included 46 patients in the Western medicine (WM) treatment group and 84 patients in the WM + Cinobufacini group. Patients in the WM + Cinobufacini group received a combination of Western medicine and oral administration of Cinobufacini Capsules/Tablets. In contrast, patients in the WM treatment group only underwent Western medicine. We gathered demographic characteristics such as age, gender, Child-Pugh classification, as well as information including Barcelona Clinic Liver Cancer (BCLC) stage, tumor size, vascular invasion, and alpha-fetoprotein (AFP) levels. We then compared the 1-year, 3-year, and 5-year overall survival (OS) and progression-free survival (PFS) between the two groups, as well as the OS and PFS for patients in the WM + Cinobufacini group who had taken the medication for different cumulative durations.

### Cell viability assays

The cell counting kit-8 (CCK-8) and CellTiter-Glo^®^ luminescent assays were carried out to evaluate the cell viability. Briefly, HCCLM3 or HepG2 cells (7 × 10^3^ cells/well) were seeded in 96-well growth-medium plate for 24 h. BF (Chengdu MUST Bio-technology Co., Ltd, A0375) was diluted with dimethyl sulfoxide (DMSO, MedChemExpress, HY-Y0320), and the cells were treated with BF (0.039, 0.078, 0.156, 0.313, 0.625, 1.25, 2.5, 5, 10, 20 µM) for another 24 h. For the CCK-8 assay, the cells were cultured with CCK-8 reagent (APExBIO Technology, K1018) for 30 min. The absorbance value at the wavelength of 450 nm were measured. For the CellTiter-Glo^®^ luminescent cell viability assay, the cells were cultured in 96-well white plates and treated with BF. Then, the CellTiter-Glo reagent (Titan, C8056S) was added in each well for 10 min. The luminescence was recorded using Microplate Reader (Tecan, Infinite 200Pro).

### Cloning formation assay

HCCLM3 and HepG2 cells (6 × 10^2^ cells/well) were seeded in a 6-well plate for 24 h, then treated with BF (50, 100, 200 nM) or sorafenib (200 nM) for 2–3 weeks. Cells were stained with crystal violet (Beyotime, C0121). Representative images were captured.

### Flow cytometry

HCCLM3 and HepG2 cells (2.5 × 10^5^ cells/well) were seeded in 6-well plate and treated with BF (50, 100, 200 nM) or sorafenib (200 nM) for 24 h. After washing with PBS, cells were stained using Alexa Fluor-conjugated Annexin V and propidium iodide (PI) (4 A Biotech, FXP018Pro-100) according to the manufacturer`s instructions, followed by a flow cytometric assay (Beckman, FACSCanto II). Raw data were analyzed by FlowJo software.

### Network pharmacological analysis

Use the PubChem database (http://pubchem.ncbi.nlm.nih.gov) to retrieve the 3D chemical structure of BF. Subsequently, employ the PharmMapper database (www.lilab-ecust.cn/pharmmapper/) to identify the targets of BF. Next, utilize the UniProt database (https://www.uniprot.org/) to convert the UniProt IDs of these targets into corresponding gene names. Conduct searches in the GeneCards database (www.genecards.org/), the DisGeNET disease database, and the Online Mendelian Inheritance in Man (OMIM) database using “Hepatocellular carcinoma” as the search keyword to obtain disease-related targets. Finally, use the VENNY 2.1 software (http://bioinfogp.cnb.csic.es/tools/venny/) to determine the intersection genes between the two sets of targets (the drug-acting targets and the disease-related targets).

Based on the intersection genes between BF and HCC, the STRING database online tool (http://string-db.org/) was utilized to construct a protein-protein interaction (PPI) network model. First, the “Multiple Proteins” option was selected, and the intersection genes were entered. The protein species was set as “Homo sapiens”, and the minimum interaction threshold was configured as “highest confidence (0.900)”. As a result, the PPI network and relevant files were obtained (with the minimum required interaction score set to highest confidence 0.900). The Cytoscape software (version 3.7.2) was then employed for network visualization. The cytoHubba plugin was used for topological structure analysis to calculate the degree and betweenness centrality of each node. Consequently, a BF-HCC-target protein regulatory network was established. The network was analyzed based on the values of degree, betweenness, node, and edge to identify key genes. Finally, the Gene Ontology (GO) term database (https://www.geneontology.org/) and the Kyoto Encyclopedia of Genes and Genomes (KEGG) pathway database (https://www.genome.jp/kegg/pathway) were applied to perform biological process enrichment analysis and KEGG pathway enrichment analysis on the key genes.

### Molecular docking

The 3D structures of the target proteins of interest were downloaded from the RCSB Protein Data Bank (PDB) (https://www.rcsb.org/). Water and impurities were then removed using PyMOL software. The 2D structure file of BF in sdf format was obtained from the PubChem database, and it was converted into a 3D structure using Chem3D software. AutoDockTools 1.5.6 software was employed to process both the receptor and the ligand. The processed files were saved in the .pdbqt format. An active-site box centered on the original ligand was set up. After obtaining the center coordinates of the box (center_x, center_y, center_z) and its size (size_x, size_y, size_z), AutoDock Vina software was run to simulate molecular docking. Finally, Discovery Studio 2021 Client software was utilized to visualize the molecular docking results between BF and the target protein.

### HCC tissue microarray

The HCC tissue microarray was sourced from Shanghai Outdo Biotech Co., Ltd. (Batch number: HLivH028PG01; array number: XT16-019). After fixation, dehydration, clearing, wax infiltration, and sectioning, the sections were subjected to hematoxylin-eosin (HE) staining. Subsequently, pathological changes were observed under an optical microscope. Immunohistochemical experiments were conducted to verify the expression of key genes in human HCC tissues.

### Plasmid transfection assay

Lipofectamine 3000 liposome transfection reagent was used to transfect the EGFR-overexpressing plasmid (pCDNA3.1-EGFR) and the empty vector (pCDNA3.1) into HepG2 and HCCLM3 cells, respectively. 6 h after transfection, the fresh culture medium was replaced, and the cells were continuously cultured for another 48 h. Subsequently, the cells were passaged into a selection medium containing G418. The surviving cells were diluted and seeded into 96-well plates for culture until monoclonal colonies formed. The expression levels of EGFR protein were detected by western blot, and stable EGFR-overexpressing monoclonal HepG2 and HCCLM3 cell lines were screened out.

### Immunoblotting

Cells or tissues were lysed with RIPA lysis buffer containing protease inhibitor for protein extraction. Standard western blotting assay was performed to analyze protein expression. The indicated primary antibodies were used for assay, and membrane was detected by using chemiluminescent HRP substrate (Millipore) in automatic chemiluminescence image analysis system (MiniChemi610, Sensi Saizhi). The intensity of bands was quantified using Image J software. The antibodies used for immunoblotting were as follows: Phospho-EGFR antibody (p-EGFR, AF3045), p-MEK1/2 antibody (AF8035), p-RAF antibody (AF3065), p-ERK1/2 (AF1015), EGFR antibody (AF6043), MEK1/2 antibody (AF6385), and Goat Anti-Rabbit IgG (H + L) Fluor594-conjugated (S0006) were bought from Affinity Bioscience. RAF1 antibody (66592-1-Ig), Pan RAS antibody (60309-1-Ig), ERK1/2 antibody (51068-1-AP), GRB2 antibody (10254-2-AP), GAPDH antiboby (60004-1-Ig), Lamin B1 (12987-1-AP), Multi-rAb HRP-Goat anti-mouse recombinant secondary antibody (RGAM001), and Multi-rAb HRP-Goat anti-rabbit recombinant secondary antibody (RGAR001) were from Proteintech.

### Immunofluorescence (IF) staining

Cells were seeded on confocal dishes and treated with BF. Next, cells were fixed with 4% paraformaldehyde and incubated with 0.1% Triton X-100. After blocked with 10% BSA and incubated with primary antibodies overnight at 4 ℃, cells were then incubated with fluorescence-conjugated secondary antibodies and subsequently counterstained with DAPI. Images were taken using a fluorescence confocal microscopy.

### Mouse subcutaneous transplanted tumor model

Forty male 6-week-old C57BL/6 mice were obtained from Beijing Vital River Laboratory Animal Technology Co., Ltd. All animals were maintained under specific pathogen-free (SPF) conditions in the Experimental Animal Center of Chengdu University of Traditional Chinese Medicine. Housing conditions included controlled temperature (22 ± 2 °C), relative humidity (50%), and a 12-hour light/dark cycle with ad libitum access to standard chow and water. Animal experiments were performed in accordance with the animal experimental protocol approved by the Institutional Animal Care and Use Committee of Chengdu University of Traditional Chinese Medicine. Following a 1-week acclimation period, subcutaneous xenograft models were established by inoculating 2 × 10^6^ Hepa1-6 cells (suspended in 200 µL PBS) into the right axillary area of each mouse. The animals were then randomly assigned to five experimental groups (*n* = 8 per group): Vehicle control group (receiving 0.2 mL saline via oral gavage), sorafenib group (30 mg/kg/day, oral gavage), BF treatment groups (0.5, 1.0, and 1.5 mg/kg/day via intraperitoneal injection). All administrations were carried out with a volume of 0.2 mL, once daily, for a duration of 21 consecutive days. The body weight, food consumption, water intake, and tumor volume of the mice were measured and recorded every alternate day. After the final administration, all mice were fasted overnight but allowed free access to water. The following day, mice were euthanized via cervical dislocation followed by eyeball exenteration, and serum/tumor specimens were harvested for biochemical and histopathological analyses.

### Mouse orthotopic transplanted tumor model

Thirty male 6-week-old BALB/C-nude mice were obtained from Beijing Vital River Laboratory Animal Technology Co., Ltd. All animals were maintained under specific pathogen-free (SPF) conditions in the Experimental Animal Center of Chengdu University of Traditional Chinese Medicine. Housing conditions included controlled temperature (22 ± 2 °C), relative humidity (50%), and a 12-hour light/dark cycle with ad libitum access to standard chow and water. Animal experiments were performed in accordance with the animal experimental protocol approved by the Institutional Animal Care and Use Committee of Chengdu University of Traditional Chinese Medicine (2024093). After 1 week of adaptive feeding, an orthotopic HCC model was established in mice. Briefly, exponentially growing HepG2-Luc cells were harvested and resuspended in sterile PBS (2 × 10^7^ cells/mL). The cell suspension was mixed 1:1 (v/v) with Corning Matrigel immediately prior to implantation. Under microscopic guidance, 200 µL of the cell-Matrigel mixture (containing 2 × 10^6^ cells) was orthotopically injected into the left hepatic lobe of each mouse. The drug administration protocols (dosage, route, and schedule) remained consistent with previously described experimental parameters. Finally, tumor growth was monitored via in vivo bioluminescence imaging after BF treatment. The mice were euthanized via cervical dislocation followed by eyeball exenteration, and serum/liver tissues were harvested for biochemical and histopathological analyses.

### Measurement of alpha-fetoprotein (AFP)

Serum was assayed for AFP (ml063401, Shanghai Enzyme-linked Biotechnology Co., Ltd.) in accordance with the manufacturer’s directions.

### Tissues hematoxylin and eosin (HE) staining

Histopathological evaluation was performed on liver/tumor tissues processed through a standardized protocol, including fixation, paraffin embedding, microtome sectioning, and HE staining. The stained sections were digitally scanned using a high-resolution slide scanner and subsequently analyzed with CaseViewer software for quantitative histopathological assessments.

### Tissue immunohistochemistry (IHC) staining

IHC was performed to determine the expression of p-EGFR, p-RAF, p-MEK1/2, and p-ERK1/2 in mouse liver and tumor. Paraffin-embedded tissue section was stained by IHC, followed by scanning with the digital pathological microtome.

### Tissue IF staining

For tissue IF, slides were deparaffinized in xylene, rehydrated in ethanol, incubated with 0.3% hydrogen peroxide, antigen retrieved with citrate buffer, and blocked with 5% BSA. Primary antibodies (p-EGFR, p-ERK1/2, p-RAF, p-MEK1/2) were incubated for 60 min in a humidified chamber at 37 ℃, followed by incubation with the corresponding secondary horseradish peroxidase-conjugated antibody. Finally, the slides were incubated with DAPI solution at 37 ℃ for 10 min in the dark. Images were detected and captured using the CaseViewer software.

### Statistical analysis

Statistical analyses of clinical retrospective analysis were performed using SPSS version 17.0 and SAS version 9.4. Categorical variables were summarized as frequencies and percentages, with between-group comparisons analyzed using chi-square tests or Fisher’s exact tests as appropriate. For continuous variables, normally distributed data were presented as mean ± standard deviation (SD) and compared using ANOVA, while non-normally distributed data were expressed as median (Q1, Q3) with between-group differences assessed via Wilcoxon rank-sum tests. PSM was implemented between the WM + Cinobufacini group and the WM group using the nearest neighbor matching algorithm with a caliper width of 0.25, achieving a 1:2 matching ratio. Survival analyses included both univariate and multivariate Cox proportional hazards regression models. Cumulative OS and PFS were estimated using Kaplan-Meier methodology, with between-group comparisons performed through log-rank tests. All statistical tests were two-sided, and p-values < 0.05 were considered statistically significant.

Statistical analyses of basic experiments were conducted using Prism 9 (GraphPad Software). All datasets are presented as mean ± SD. Comparative analyses employed unpaired Student’s t-test for two-group comparisons or one-way ANOVA with Dunnett’s post hoc test for multi-group comparisons. Significance threshold was defined as *P* < 0.05.

## Results

### Survival benefit of cinobufacini in HCC patients

To investigate therapeutic outcomes of Cinobufacini in HCC, 204 patients meeting predefined eligibility criteria were enrolled and stratified into two cohorts: 53 patients received WM therapy, while 151 patients were assigned to WM + Cinobufacini therapy. Baseline clinical profiles were systematically characterized through demographic parameters (age, sex), biochemical markers (serum albumin [ALB], total bilirubin [TBIL], prealbumin [PALB], microalbuminuria [MALB], prothrombin time [PT], international normalized ratio [INR], prothrombin time activity [PTA], aspartate aminotransferase [AST], alanine aminotransferase [ALT]), serum tumor biomarkers (alpha-fetoprotein [AFP], carbohydrate antigen 19 − 9 [CA19-9], carcinoembryonic antigen [CEA]), and disease staging systems (BCLC, Child-Pugh stage). Initial comparative analyses revealed significant intergroup disparities in ALB (*P* < 0.001), TBIL (*P* < 0.001), PT (*P* = 0.025), INR (*P* = 0.038), AST (*P* = 0.002), and CA19-9 levels (*P* = 0.014) (Supplementary Table 1). To mitigate confounding biases arising from baseline heterogeneity, we performed 1:2 nearest-neighbor PSM using covariates demonstrating pre-matching disparities. This process yielded balanced cohorts: 46 patients in the WM group and 84 patients in the WM + Cinobufacini group. Post-matching validation confirmed equivalent baseline distributions across all previously imbalanced variables (all *P* > 0.05) (Table 1), achieving adequate comparability for subsequent outcome analysis.

**Table 1:** Demographic parameters, biochemical markers, and disease staging in two study groups after PSM

Characteristics	WM therapy	WM + Cinobufacini therapy	Statistics	*P* value
	(*n* = 46)	(*n* = 84)		
Age (year)	60.96 ± 13.95	61.17 ± 11.27	-0.09(t)	0.926
Sex				
Female	13(28.26)	14(16.67)	2.43(Chi-square)	0.119
Male	33(71.74)	70(83.33)		
Child-Pugh Stage				
A	14(30.43)	24(28.57)	0.19(Chi-square)	0.910
B	19(41.30)	38(45.24)		
C	13(28.26)	22(26.19)		
BCLC Stage				
A	0(0.00)	0(0.00)	0.19(Chi-square)	0.912
B	22(47.83)	40(47.62)		
C	14(30.43)	28(33.33)		
D	10(21.74)	16(19.05)		
ALB (g/L)	29.74 ± 8.89	30.55 ± 9.87	-0.46(t)	0.644
TBIL (µmol/L)	30.4(18.1,122.2)	29.6(17.5,59.7)	0.99(Z)	0.323
PALB (g/L)	97.0(47.1,148.9)	68.0(30.2,127.9)	1.86(Z)	0.063
MALB (mg/24 h)	0.2(0.2,0.2)	0.2(0.2,0.2)	. (Z)	.
PT (s)	16.27 ± 3.92	15.87 ± 4.46	0.51(t)	0.612
INR	1.23 ± 0.28	1.20 ± 0.33	0.61(t)	0.544
PTA (%)	82.65 ± 21.66	79.73 ± 22.62	0.72(t)	0.475
AST (U/L)	97.5(58.0,187.0)	72.0(36.0,128.0)	1.37(Z)	0.172
ALT (U/L)	46.5(20.0,80.0)	41.5(20.0,95.0)	0.22(Z)	0.828
AFP (ng/mL)	70.4(7.4,825.2)	72.3(6.4,1210.0)	-0.44(Z)	0.661
CA19-9 (U/mL)	66.6(18.4,258.3)	32.3(16.9,102.4)	1.19(Z)	0.233
CEA (ng/mL)	3.0(1.9,5.7)	3.5(2.1,5.6)	-0.43(Z)	0.666
1 year’s progress				
No progress	15(32.61)	32(38.10)	0.39(Chi-square)	0.534
Progress	31(67.39)	52(61.90)		
3 year’s progress				
No progress	3(6.52)	3(3.57)	0.24(Fisher)	0.665
Progress	43(93.48)	81(96.43)		
5 year’s progress				
No progress	0(0.00)	2(2.38)	0.42(Fisher)	0.539
Progress	46(100.0)	82(97.62)		
1 year’s end				
Survival	25(54.35)	54(64.29)	1.23(Chi-square)	0.267
Death	21(45.65)	30(35.71)		
3 year’s end				
Survival	6(13.04)	21(25.00)	2.58(Chi-square)	0.108
Death	40(86.96)	63(75.00)		
5 year’s end				
Survival	2(4.35)	6(7.14)	0.26(Fisher)	0.712
Death	44(95.65)	78(92.86)		

Note: Normal reference ranges: ALB 35–55 g/L; TBIL 0–21 µmol/L; PALB 20–40 g/L; MALB 0–30 mg/24 h; PT 9.6–13 s; INR 0.8–1.2; PTA 70–130%; AST 15–40 U/L; ALT 9–50 U/L; AFP < 25 ng/mL; CA19-9 < 37 U/mL; CEA < 5 ng/mL

**Fig. 1:**
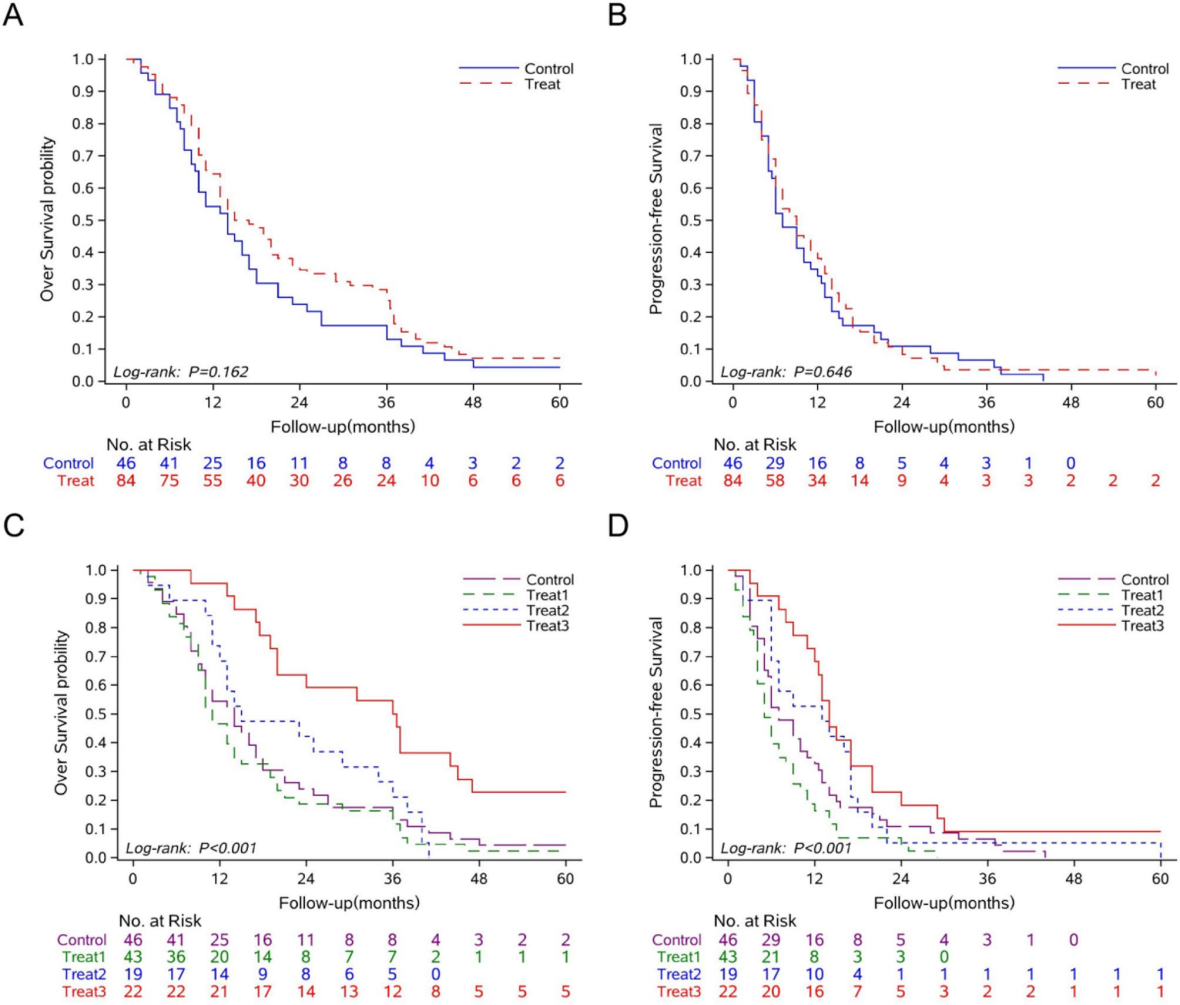
The survival benefit observed in HCC patients receiving Cinobufacini. (**A**, **B**) Comparison of 5-year OS (**A**) and PFS (**B**) between the two groups of patients after PSM. Control, Western medicine (WM) therapy. Treat, WM + Cinobufacini combination therapy. (**C**,** D**) Comparison of OS (**C**) and PFS (**D**) with different cumulative medication durations. Control, WM therapy. Treat1, cumulative treatment duration of Cinobufacini: <30 days. Treat2, cumulative treatment duration of Cinobufacini: 30–60 days. Treat3, cumulative treatment duration of Cinobufacini: >60 days

After PSM adjustment, the WM therapy group demonstrated 1-, 3-, and 5-year OS of 54.35%, 13.04%, and 4.35%, respectively, compared to 64.29%, 25.00%, and 7.14% in the WM + Cinobufacini combination therapy group (Fig. 1A). Although these differences did not reach statistical significance (all *P* > 0.05), the consistent numerical superiority of combination therapy across all timepoints suggests a potential prognostic benefit associated with Cinobufacini supplementation. PFS outcomes exhibited a more complex pattern, with 1-year PFS of 32.61% (WM) vs. 38.10% (WM + Cinobufacini) and 3-year PFS of 6.52% vs. 3.57%. Notably, the 5-year PFS analysis revealed complete divergence, with 0% survival in the WM group compared to 2.38% in the combination group (Fig. 1B). The observed survival trends warrant further investigation in larger prospective trials to confirm whether Cinobufacini confers measurable clinical benefits when added to standard WM regimens for HCC.

To delineate the exposure-response relationship between Cinobufacini therapy and clinical outcomes, we stratified the combination therapy cohort into 3 subgroups based on cumulative treatment duration of Cinobufacini: short-term (< 30 days), intermediate-term (30–60 days), and prolonged exposure (> 60 days). Following PSM adjustment, survival analyses revealed significant dose-dependent survival benefits across all exposure subgroups compared to the WM therapy group (*P* < 0.001 for both OS and PFS, Fig. 1C, D). The exploratory analysis of our observational data suggests a potential relationship between prolonged Cinobufacini exposure and favorable clinical outcomes in HCC management, with preliminary evidence hinting at a possible duration-dependent therapeutic response. On the other hand, this subgroup analysis was post hoc and hypothesis-generating rather than confirmatory. While intriguing, these results require validation in prospective studies with predefined endpoints to mitigate the risk of false-positive associations and establish optimal integration strategies for Cinobufacini in evidence-based HCC treatment paradigms.

### BF inhibits cell proliferation and induces apoptosis in HepG2 and HCCLM3

**Fig. 2:**
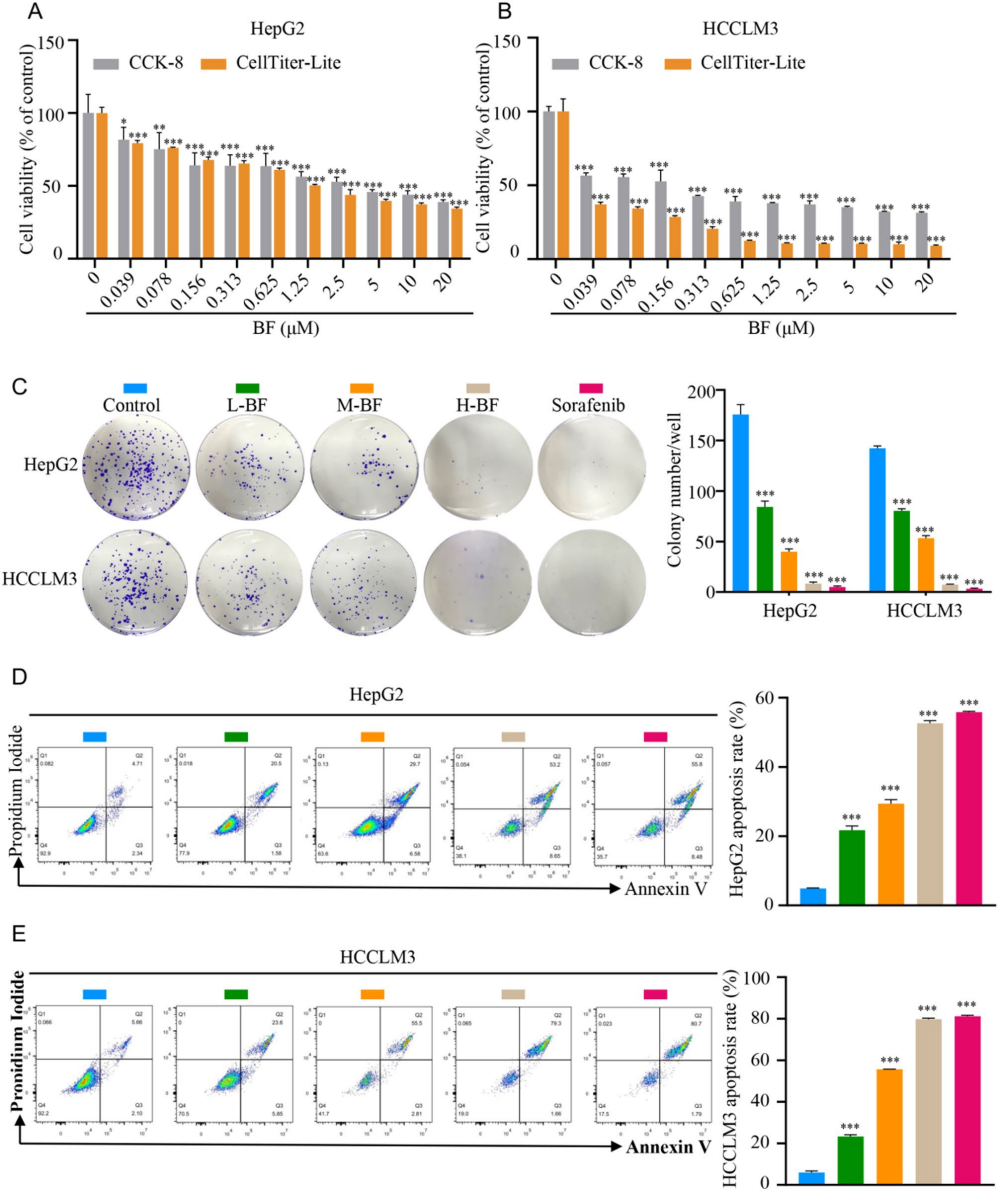
BF inhibits cell proliferation and induces apoptosis in HepG2 and HCCLM3. (**A**,** B**) The effect of BF on cell proliferation through CCK-8 and CellTiter-Lite assays in HepG2 (**A**) and HCCLM3 (**B**). (**C**) The influence of BF on the clone formation of HepG2 and HCCLM3. (**D**,** E**) The effect of BF on the apoptosis of HepG2 (**D**) and HCCLM3 (**E**). Data are expressed as mean ± SD from three independent experiments with biological duplicates. ^*^P < 0.05, ^**^P < 0.01, and ^***^P < 0.001 compared to the Control group

To evaluate the therapeutic potential of BF, we conducted comprehensive functional assays in vitro models. CCK-8 viability assays revealed robust, concentration-dependent inhibition of proliferation in both HepG2 and HCCLM3 following BF exposure (Fig. 2A, B). These findings were corroborated by complementary ATP production assays using CellTiter-Lumi™, which demonstrated dose-responsive reductions in metabolic activity (as measured by luminescence intensity) across both cell lines (Fig. 2A, B). Clonogenic survival assays further validated these antiproliferative effects, with BF treatment resulting in significant dose-dependent reductions in colony formation efficiency compared to vehicle controls in both HCCLM3 and HepG2 (Fig. 2C). Flow cytometric analysis revealed BF-induced apoptosis in a concentration-dependent manner in HCCLM3 and HepG2, as evidenced by annexin V/PI staining (Fig. 2D, E). At 200 nM concentrations, BF induced apoptotic rates of 52.60 ± 1.06% in HepG2 and 79.73 ± 0.21% in HCCLM3, comparable to the reference compound sorafenib (55.83 ± 0.21% and 81.20 ± 0.35%, respectively). Collectively, these results demonstrate that BF exerts potent antiproliferative and pro-apoptotic effects against HCC cells. The observed concentration-dependent responses suggest BF may represent a promising therapeutic candidate for HCC treatment, warranting further investigation into its molecular mechanisms and translational potential.

### PPI network and key target genes of BF in the treatment of HCC

**Fig. 3:**
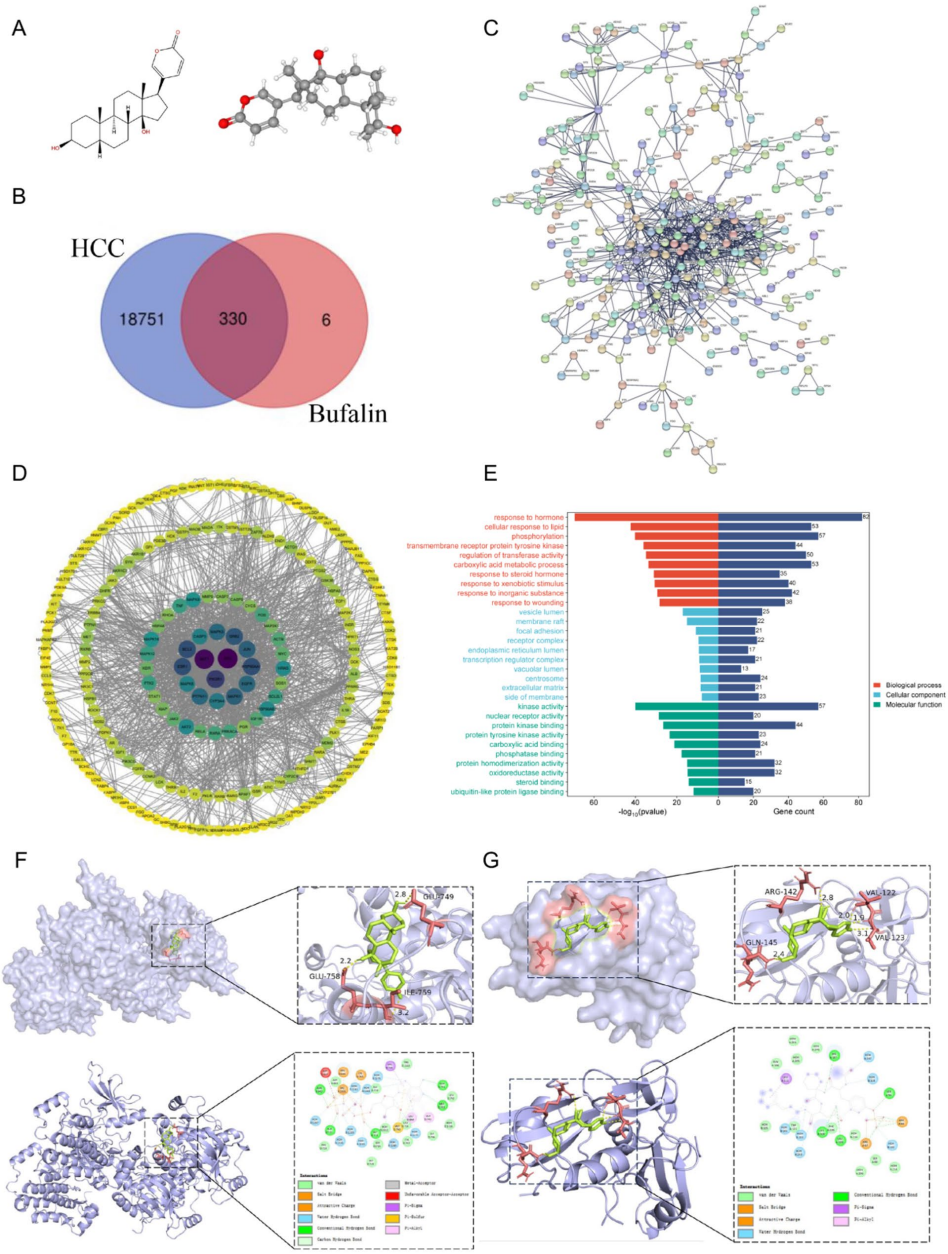
Key target genes and molecular docking of BF in the treatment of HCC. (**A**) The 2D and 3D chemical structures of BF. (**B**) The intersection gene of BF and HCC. (**C**) BF-HCC PPI network diagram. (**D**) The protein interaction network diagram of key targets in BF-HCC. (**E**) Difference analysis of biological process, molecular function, and cellular component. (**F**) Diagram of the docking mode between BF and EGFR. (**G**) Diagram of the docking mode between BF and GRB2

To delineate the molecular underpinnings of BF’s therapeutic potential against HCC, we employed an integrative systems pharmacology framework. Initially, two-dimensional (2D) and three-dimensional (3D) chemical structures of BF were retrieved from the PubChem database (Fig. 3A). A pharmacophore mapping strategy using PharmMapper yielded 300 candidate targets (the top 300 targets ranked in descending order by the standardized fit score, ID: 240517083752), which were systematically augmented with functional annotations from the Comparative Toxicogenomics Database (CTD) and Search Tool for Interactions of Chemicals (STITCH). Following data integration and removal of redundant entries, a final set of 336 BF-associated targets was established.

For disease-specific target acquisition, a multi-database mining strategy was implemented using GeneCards, DisGeNET, and the Online Mendelian Inheritance in Man (OMIM) repository. This comprehensive approach initially identified 30,167 disease-associated targets, which was refined to 19,081 unique entries after deduplication and data harmonization. VENNY 2.1 software was employed to identify overlapping targets between BF-related and HCC-related gene sets, revealing 330 intersection targets (Fig. 3B). This integrative bioinformatics approach established the molecular basis for subsequent network pharmacology analysis and functional validation studies.

PPI networks for BF-target genes and HCC-related genes were constructed using the STRING database, yielding 124 overlapping critical genes (Fig. 3C). The resulting PPI network was visualized using Cytoscape 3.7.2 software (Fig. 3D). Network topology analysis based on node degree and betweenness centrality metrics identified 15 hub targets, including SRC, AKT1, PIK3R1, HSP90AA1, ESR1, BCL2, GRB2, MAPK1, JUN, EGFR, MAPK3, PTPN11, CYP3A4, MAPK8, and CASP3. According to the significance degree of the P value, further GO enrichment analysis and KEGG pathway analysis were conducted. The functional enrichment bar chart was drawn, and the bubble chart was selected for visualization processing (Supplementary Fig. 1A, B). Subsequent biological interpretation focused on HCC-relevant biological processes, cellular components, and molecular functions, mainly manifested in inflammatory response, hormonal response, regulation of transcription factors, regulation of gene expression, cytokine activity, molecular structure of adhesion plaques and protein binding activity, etc. (Fig. 3E). These findings suggest BF exerts multimodal therapeutic effects through modulation of key oncogenic signaling hubs and tumor microenvironment interactions.

### Molecular docking results

To investigate potential molecular interactions, virtual screening was performed by molecular docking of candidate core targets with BF. Targets exhibiting binding energies ≤ -5.0 kcal/mol were prioritized for subsequent analysis. The molecular docking results were visualized using the Discovery Studio 2021 Client software. It was found that the binding energies of EGFR, GRB2, SRC, MAPK1 and BF docking were all less than − 5 kcal/mol. Among the evaluated targets, EGFR (PDB structure: 7JXQ) exhibited the binding affinity with BF, achieving a docking score of -7.7 kcal/mol (Fig. 3F), while GRB2 (PDB structure: 6WM1) demonstrated a comparable interaction with a binding energy of -8.5 kcal/mol (Fig. 3G). These values indicate highly favorable ligand-receptor complex formation, with both targets exceeding the predefined screening threshold of ≤ -5.0 kcal/mol. Based on these findings, subsequent experimental validation will focus on elucidating the regulatory mechanisms of EGFR and GRB2 expression, as these targets demonstrate both structural compatibility with BF and biological relevance to the investigated pathway.

### High expression of EGFR and GRB2 in HCC tissue microarray

**Fig. 4:**
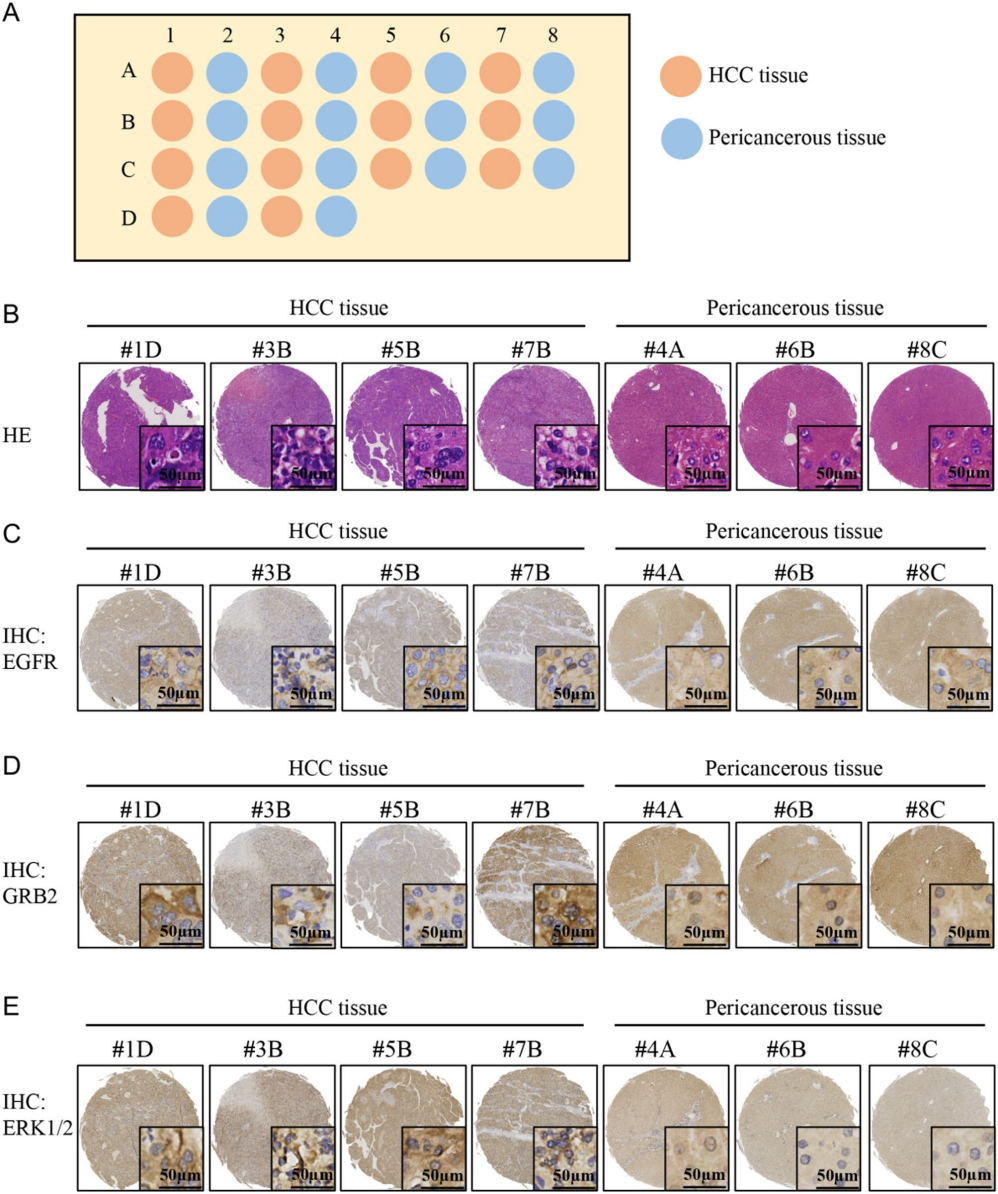
HE staining, and EGFR/GRB2/ERK1/2 expression in HCC tissue microarray. (**A**) Pattern diagram of HCC tissue microarray. (**B**) IHC staining with EGFR-specific antibody to detect EGFR expression in tissue microarray. (**C**) IHC staining with GRB2-specific antibody to detect GRB2 expression in tissue microarray. (**D**) IHC staining with ERK1/2-specific antibody to detect ERK1/2 expression in tissue microarray

To experimentally validate the translational relevance of our computational predictions, we performed IHC analysis on an HCC tissue microarray (Fig. 4A) containing matched tumoral (*n* = 14 cases) and adjacent non-tumorous liver (ANT) specimens (*n* = 14 cases). HE staining (Fig. 4B, Supplementary Fig. 2A) revealed that ANT tissues exhibited normal histological architecture, characterized by polygonal hepatocytes with large, round, and centrally located nuclei. The cytoplasmic borders were well-defined, with no evidence of atypia or pathological lesions observed. In contrast, HCC tissues displayed dense architectural arrangement with enlarged nuclei exhibiting irregular contours. Extensive infiltration of tumor cells with infiltrative growth patterns was noted, accompanied by stromal desmoplasia. The overall pathological grade was classified as Edmondson-Steiner grade II-III, reflecting moderate-to-poor differentiation and aggressive biological behavior. EGFR immunostaining revealed striking differential expression patterns: ANT tissues exhibited near-absent staining, whereas HCC lesions demonstrated moderate-to-strong cytoplasmic/membranous positivity with intense perinuclear accentuation in malignant hepatocytes (Fig. 4C, Supplementary Fig. 2B). GRB2 immunoreactivity showed complementary expression dynamics, with ANT tissues exhibiting negative staining and HCC samples displaying robust cytoplasmic staining characterized by diffuse granular deposition in the perinuclear region (Fig. 4D, Supplementary Fig. 2C), and ERK1/2 exhibited the same effect (Fig. 4E, Supplementary Fig. 2D). These findings corroborate our network pharmacology predictions and molecular docking simulations, which demonstrated favorable BF binding to EGFR and GRB2 catalytic domains. Integrating these data with our previous systems-level analysis, we propose the mechanism of BF action: inhibition of oncogenic drivers EGFR/MAPK axis. Future investigations will focus on elucidating the functional significance of these interactions through in vitro/in vivo validation studies.

### BF inhibits EGFR-mediated RAS/RAF/MEK/ERK signaling pathway activation

**Fig. 5:**
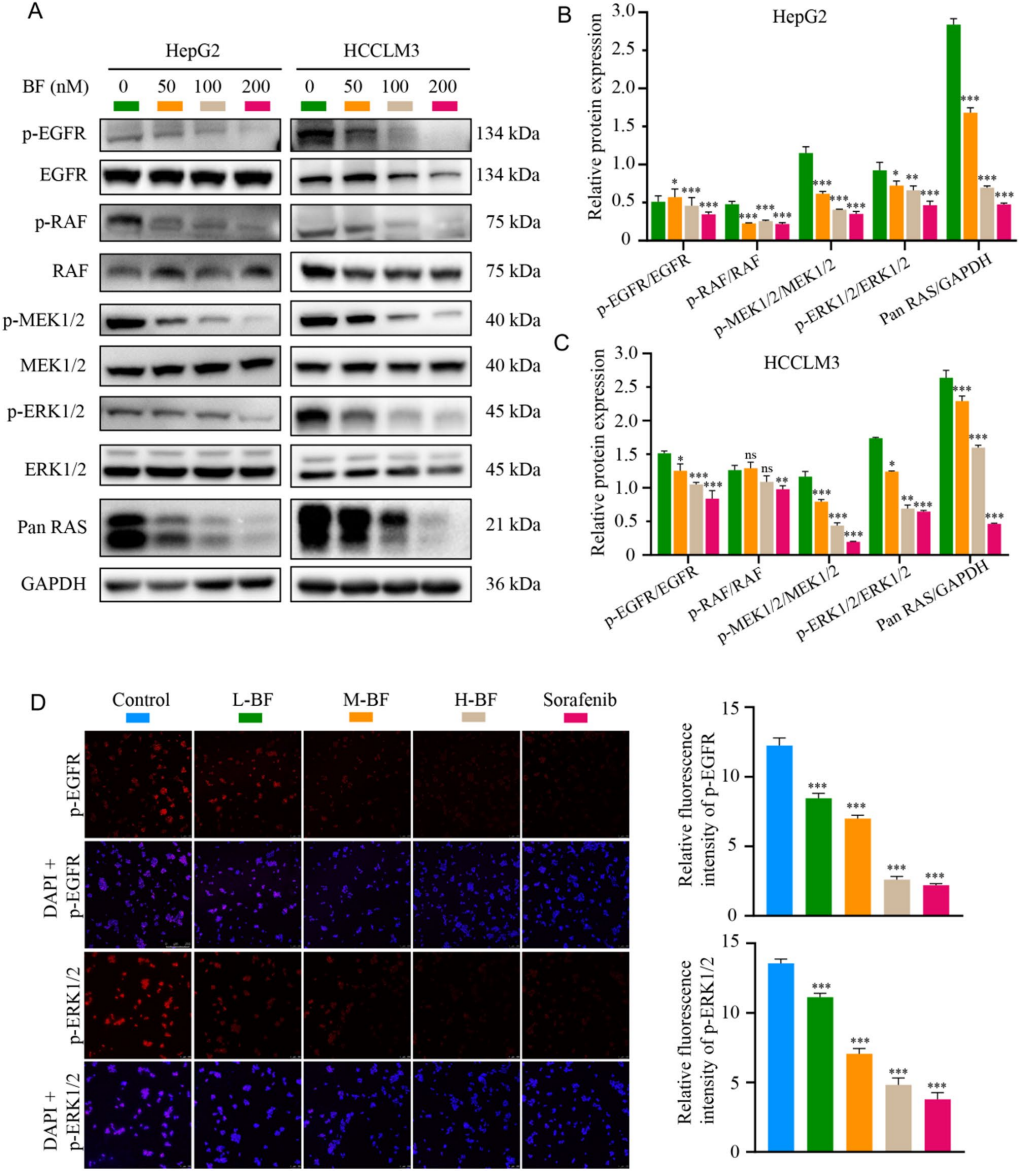
BF inhibits the activation of EGFR-mediated RAS/RAF/MEK/ERK pathway. (**A**) Immunoblotting of p-EGFR, EGFR, p-RAF, RAF, p-MEK1/2, MEK1/2, p-ERK1/2, ERK1/2, and Pan RAS protein expression in HepG2 and HCCLM3. (**B**, **C**) Relative protein expression of p-EGFR/EGFR, p-RAF/RAF, p-MEK1/2/MEK1/2, p-ERK1/2/ERK1/2, and Pan RAS/GAPDH in HepG2 (**B**) and HCCLM3 (**C**). (**D**) IF staining of p-EGFR and p-ERK1/2 in HepG2. (**E**, **F**) Relative fluorescence intensity of p-EGFR (**E**) and p-ERK1/2 (**F**) in HepG2. Data are expressed as mean ± SD from three independent experiments with biological duplicates. ^*^P < 0.05, ^**^P < 0.01, and ^***^P < 0.001 compared to the Control group

These previous findings suggest that BF mediates its antitumor effects against HCC through dual inhibition of EGFR activation and subsequent attenuation of downstream RAS-RAF-MEK-ERK signaling cascade activation. This pathway-specific suppression disrupts oncogenic signal transduction, thereby inhibiting proliferative and pro-survival cues critical for HCC maintenance. HepG2 and HCCLM3 cells were treated with BF (50, 100, 200 nM) for 24 h. Immunoblotting revealed concentration-dependent suppression of p-EGFR, p-RAF, p-MEK1/2, p-ERK1/2, and Pan RAS protein expression in both cell lines (Fig. 5A-C). IF staining further confirmed these findings, showing progressive attenuation of p-EGFR, p-RAF, p-MEK1/2, and p-ERK1/2 immunoreactivity in BF-treated groups compared to vehicle controls (Fig. 5D-F, Supplementary Fig. 3). These observations suggest BF exerts its antiproliferative effects through dual inhibition of EGFR activation and downstream RAS-RAF-MEK-ERK pathway signaling.

**Fig. 6:**
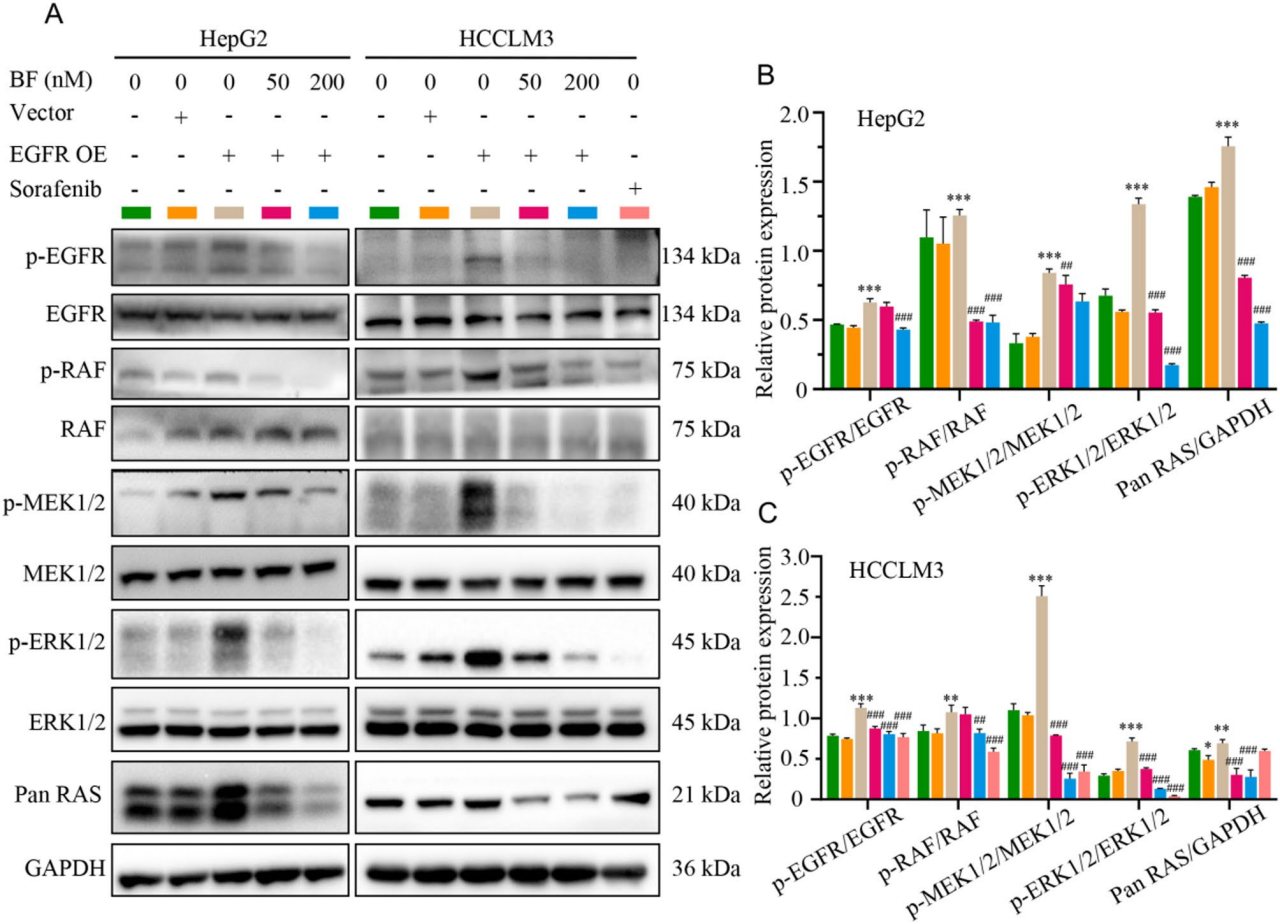
BF inhibits RAS/RAF/MEK/ERK pathway activation in EGFR overexpression system. (**A**) Immunoblotting of p-EGFR, EGFR, p-RAF, RAF, p-MEK1/2, MEK1/2, p-ERK1/2, ERK1/2, and Pan RAS protein expression in EGFR overexpression HepG2 and HCCLM3. (**B**,** C**) Relative protein expression of p-EGFR/EGFR, p-RAF/RAF, p-MEK1/2/MEK1/2, p-ERK1/2/ERK1/2, and Pan RAS/GAPDH in HepG2 (**B**) and HCCLM3 (**C**). Data are expressed as mean ± SD from three independent experiments with biological duplicates. ^*^P < 0.05, ^**^P < 0.01, and ^***^P < 0.001 compared to the Control group. ^###^*P* < 0.001 compared to the EGFR OE group

To validate the therapeutic mechanism of BF in EGFR-driven HCC, we evaluated its impact on RAS/RAF/MEK/ERK signaling using EGFR-overexpressing HepG2 and HCCLM3 cell models. Immunoblotting revealed significant upregulation of p-EGFR, p-RAF, p-MEK1/2, p-ERK1/2, and Pan RAS in EGFR-transfected cells compared to vector controls (Fig. 6A-C). Treatment with high-dose BF (200 nM) reversed this oncogenic phenotype, demonstrating concentration-dependent suppression of these proteins. These findings establish a mechanistic link between BF treatment and EGFR pathway inhibition, demonstrating that BF antagonizes HCC progression by suppressing EGFR-mediated activation of the RAS-RAF-MEK-ERK axis. The observed dose-dependent attenuation of both receptor tyrosine kinase phosphorylation and downstream effector proteins provides compelling evidence for pathway-specific therapeutic targeting. This dual inhibitory effect disrupts critical pro-survival signaling networks, offering a rational basis for BF’s antiproliferative activity in EGFR-addicted HCC subtypes.

### BF inhibits the growth of subcutaneous HCC tumors in C57BL/6 mice

**Fig. 7:**
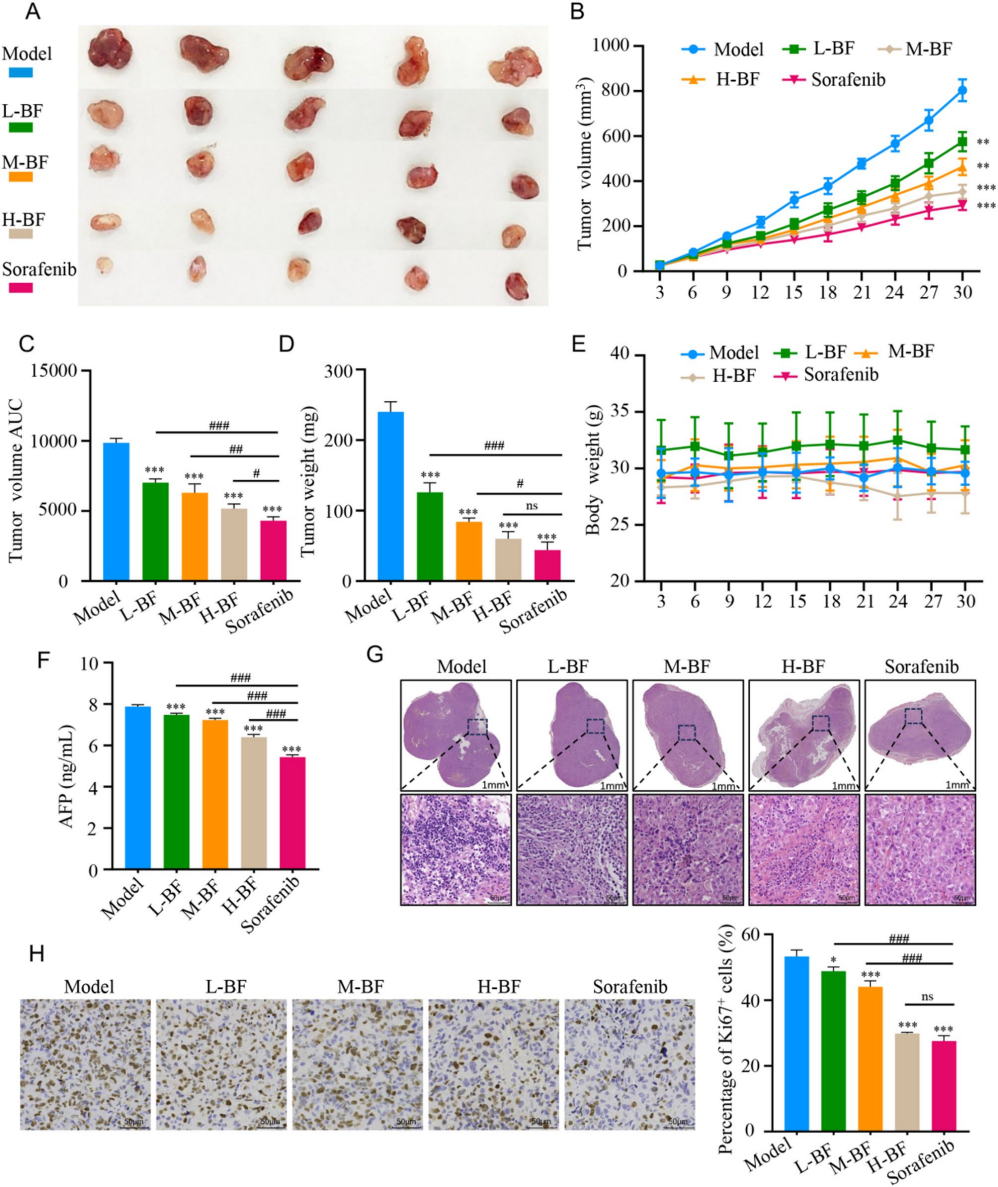
BF inhibits the growth of subcutaneous HCC tumors. (**A**) Representative figures of subcutaneous HCC tumors. (**B**-**D**) Tumor volumes (**B**) and weights (**C**, **D**) were measured at indicated times. (**E**) The curve of weight change in mice. (**F**) AFP concentration in mouse serum. (**G**) Pathological changes of tumor tissues in mice. (**H**) The expression of Ki67 in mouse liver cancer tumor tissues. Data are expressed as mean ± SD from three independent experiments with biological duplicates. ^*^P < 0.05, ^**^P < 0.01, and ^***^P < 0.001 compared to the Model group. ^#^*P* < 0.05, ^##^*P* < 0.01, and ^###^*P* < 0.001 compared to the Sorafenib group

To evaluate the in vivo antitumor efficacy of BF against HCC, we established a subcutaneous xenograft model using Hepa1-6 cells in C57BL/6. Tumor volume measurements revealed significant dose-dependent reductions in BF-treated groups compared to vehicle controls (Fig. 7A, B). High-dose BF (1.5 mg/kg) achieved comparable tumor volume suppression to sorafenib (30 mg/kg) throughout the observation period. Consistent with volumetric analysis, tumor weight assessment confirmed significant antitumor activity of BF (Fig. 7C). Area-under-the-curve (AUC) analysis of tumor growth trajectories confirmed this dose-dependent response pattern (Fig. 7D). Importantly, BF treatment did not induce systemic toxicity, as evidenced by stable body weight profiles across all dosage groups (Fig. 7E). Serum biomarker analysis further supported therapeutic efficacy, showing significant reductions in AFP levels following BF administration (Fig. 7F). These collective findings establish BF as a potent inhibitor of HCC xenograft growth with favorable safety profile and comparable efficacy to first-line sorafenib therapy.

Histopathological evaluation via HE staining revealed pronounced morphological differences between treatment groups (Fig. 7G). Vehicle-control tumors exhibited poorly differentiated architecture characterized by marked nuclear pleomorphism, hyperchromatic nuclei, and infiltrative growth patterns with extensive stromal invasion. In contrast, high-dose BF and sorafenib-treated tumors displayed improved tissue organization with loose cellular arrangements, reduced nuclear atypia, and diminished invasive margins. Immunohistochemical analysis of Ki67 proliferation index further corroborated these findings (Fig. 7H). Vehicle-control tumors demonstrated intense nuclear staining, indicative of robust proliferative activity. BF treatment induced dose-dependent suppression of Ki67 expression. Notably, high-dose BF achieved comparable antiproliferative effects to sorafenib. Staining intensity analysis confirmed this trend, with vehicle tumors showing strong positivity, moderate positivity in BF-mid, and weak positivity in BF-high/sorafenib groups. These combined histopathological and immunohistochemical assessments demonstrate that BF exerts potent antitumor effects through dual inhibition of malignant transformation and cell proliferation.

### BF inhibits RAS/RAF/MEK/ERK signaling pathway activation in tumor tissues

**Fig. 8:**
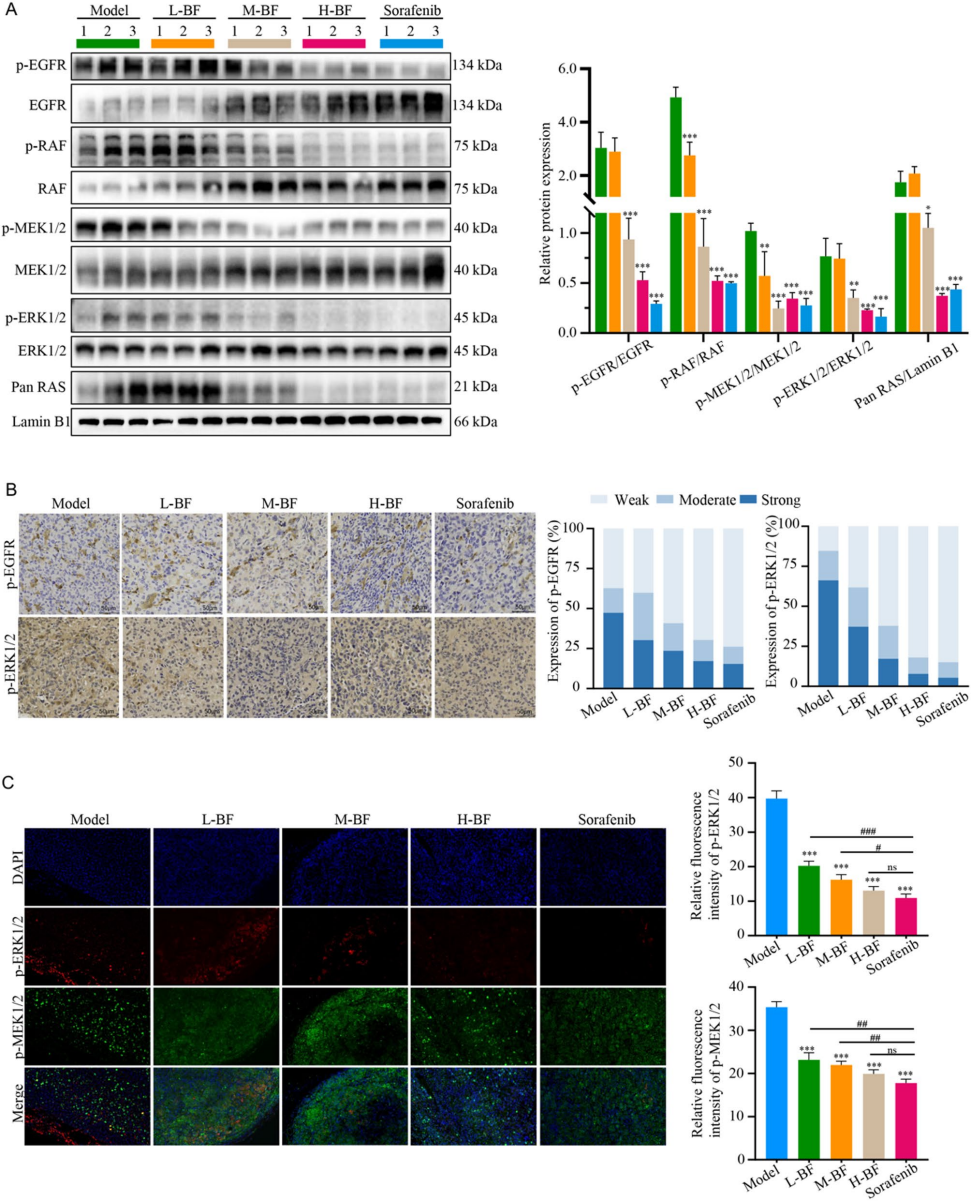
BF inhibits RAS/RAF/MEK/ERK signaling pathway activation in subcutaneous HCC tumors. (**A**) Immunoblotting of p-EGFR, EGFR, p-RAF, RAF, p-MEK1/2, MEK1/2, p-ERK1/2, ERK1/2, and Pan RAS protein expression. (**B**) Relative protein expression of p-EGFR/EGFR, p-RAF/RAF, p-MEK1/2/MEK1/2, p-ERK1/2/ERK1/2, and Pan RAS/GAPDH. (**C**) IHC staining of p-EGFR and p-ERK1/2 in subcutaneous HCC tumors. (**D**) IF staining of p-MEK1/2 and p-ERK1/2 in subcutaneous HCC tumors. Data are expressed as mean ± SD from three independent experiments with biological duplicates. ^*^P < 0.05, ^**^P < 0.01, and ^***^P < 0.001 compared to the Control group. ^#^*P* < 0.05, ^##^*P* < 0.01, and ^###^*P* < 0.001 compared to the Sorafenib group

To validate the pharmacological targeting of RAS/RAF/MEK/ERK signaling by BF, we performed comprehensive analysis of pathway activation status in tumor tissues. Immunoblotting revealed significant suppression of p-EGFR, Pan RAS, p-RAF, p-MEK1/2, and p-ERK1/2 in BF-treated groups compared to vehicle controls (Fig. 8A, B). IHC staining corroborated these findings, demonstrating dose-dependent reduction in phosphorylated protein immunoreactivity. Vehicle-control tumors exhibited intense staining for p-EGFR, p-RAF, p-MEK1/2, and p-ERK1/2 with diffuse cytoplasmic/membranous localization. BF treatment induced graded attenuation of staining intensity (Fig. 8C, Supplementary Fig. 4). IF microscopy further confirmed pathway inhibition, showing progressive reduction in mean fluorescence intensity of phosphorylated proteins. Vehicle tumors exhibited robust fluorescence signals. Notably, high-dose BF achieved comparable fluorescence reduction to sorafenib (Fig. 8D, Supplementary Fig. 5). Collectively, these multi-modal analyses establish that BF exerts its antitumor effects through dual inhibition of EGFR receptor tyrosine kinase activity and downstream RAS/RAF/MEK/ERK cascade activation. The observed dose-dependent suppression of pathway biomarkers provides compelling evidence for therapeutic targeting of this critical oncogenic signaling axis in HCC.

### BF inhibits the growth of orthotopic HCC tumors and the activation of EGFR-mediated RAS/RAF/MEK/ERK pathway in BALB/C-nude mice

**Fig. 9:**
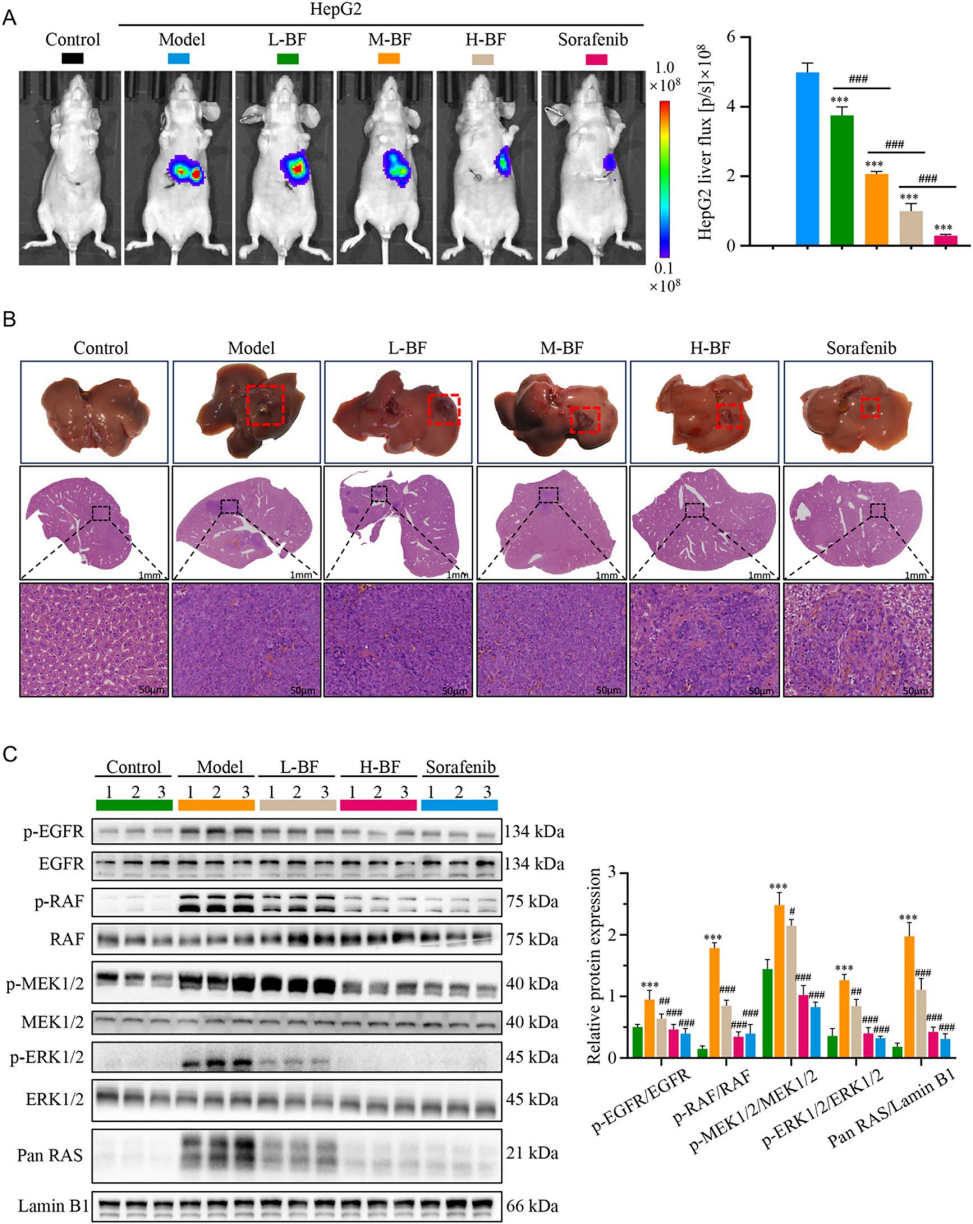
BF inhibits the growth of orthotopic HCC tumors and EGFR-mediated RAS/RAF/MEK/ERK pathway activation. (**A**) Bioluminescence images of HCC tumors in in HepG2-Luc cells-treated BALB/C-nude mice. The color scale bar depicts the photon flux emitted from tumors. (**B**) Representative images are presented at the endpoint. Tumor sites are indicated by red dotted box. Tumor sites in livers were detected by HE staining. (**C**) Immunoblotting of p-EGFR, EGFR, p-RAF, RAF, p-MEK1/2, MEK1/2, p-ERK1/2, ERK1/2, and Pan RAS protein expression. Data are expressed as mean ± SD from three independent experiments with biological duplicates. ^***^P < 0.001 compared to the Control group. ^#^*P* < 0.05, ^##^*P* < 0.01, and ^###^*P* < 0.001 compared to the Model group

To evaluate the therapeutic efficacy of BF against orthotopic HCC, we established an intrahepatic tumor model in BALB/c-nude mice using luciferase-expressing HepG2 cells (HepG2-Luc). In vivo bioluminescence imaging demonstrated progressive tumor proliferation in HepG2-Luc cells-treated animals, as evidenced by the increases in bioluminescence signal intensity (Fig. 9A). In contrast, BF-treated groups exhibited dose-dependent suppression of tumor growth compared to models (Fig. 9A). Ex vivo analysis of excised livers confirmed these imaging findings, with high-dose BF and sorafenib groups exhibiting reductions in tumor volume respectively compared to models (Fig. 9B). HE staining further demonstrated reduced tumor burden in BF-treated groups, characterized by decreased cellular density, preserved hepatic parenchyma, and attenuated stromal invasion (Fig. 9B). The observed dose-dependent reductions in bioluminescence signal and tumor volume, coupled with histopathological evidence of reduced invasiveness, provide compelling preclinical evidence supporting BF as a potential therapeutic agent for HCC.

Immunoblotting of orthotopic HCC tissues confirmed pathway-specific modulation by BF treatment. Compared to models, BF-treated tumors exhibited significant dose-dependent reductions in p-EGFR, Pan RAS, p-RAF, p-MEK1/2, and p-ERK1/2 (Fig. 9C). IHC analysis validated these mechanistic insights, revealing a concentration-dependent decline in phospho-protein expression across the EGFR-mediated RAS/RAF/MEK/ERK axis after BF treatment (Fig. 9D). Collectively, these findings demonstrate that BF exerts potent antitumor effects in orthotopic HCC through the inhibition of EGFR-mediated RAS/RAF/MEK/ERK signaling cascade activation. The observed dose-dependent suppression of pathway biomarkers, coupled with reductions in tumor burden and invasiveness, provide compelling mechanistic evidence supporting BF as a therapeutic candidate for HCC.

## Discussion

The combination of WM therapy with Cinobufacini demonstrated a trend toward improved OS in HCC patients, with prolonged administration duration correlating with enhanced survival benefits and reduced disease progression. The lack of statistical significance in the overall cohort may reflect limitations in sample size and patient heterogeneity. Given the retrospective nature of the study, potential biases, limited statistical power, and the risk of false positives due to post hoc analyses must be acknowledged. Notably, the 3-year and 5-year OS improvement observed in the treatment group warrant validation through larger multicenter prospective trials. Such studies should incorporate biomarker-driven patient stratification and longitudinal pharmacodynamic monitoring to establish definitive clinical efficacy. These preliminary findings highlight the need for phase III randomized controlled trials with standardized dosing regimens and quality-of-life assessments to fully elucidate the therapeutic value of this traditional Chinese medicine formulation in modern HCC management protocols.

We further investigated the anticancer effects and underlying mechanisms of BF, the key active component of Cinobufacini, in HCC. Through integrated network pharmacology analysis and molecular docking validation, we identified EGFR and GRB2 as primary therapeutic targets of BF in HCC treatment. Immunohistochemical evaluation of tissue microarrays revealed significantly elevated expression levels of both EGFR and GRB2 in HCC tissues compared to ANT liver tissues. The observed overexpression of these targets in malignant hepatocytes suggests a potential regulatory role in HCC pathogenesis and therapeutic response to BF intervention. Recent studies have highlighted the aberrant expression of GRB2 across multiple malignancies, including HCC, glioblastoma, colorectal carcinoma, cholangiocarcinoma, pancreatic ductal adenocarcinoma, and gastric cancer [20–22]. Emerging evidence suggests its expression level correlates with clinical prognosis and therapeutic response patterns in these malignancies. GRB2, a key molecule in the RAF/MEK/ERK pathway, mediates tumor cell proliferation, invasion, and migration [23, 24]. EGFR, a transmembrane receptor tyrosine kinase, undergoes conformational changes upon ligand binding, thereby activating its intrinsic tyrosine kinase activity and initiating downstream signaling cascades [25]. EGFR frequently exhibits overexpression or mutations across various malignancies, including HCC, non-small cell lung cancer, breast carcinoma, pancreatic adenocarcinoma, nasopharyngeal carcinoma, and head and neck squamous cell carcinoma [26–29]. Study has suggested that BF shows strong antitumor effect in cancers with high wild-type EGFR expression [30]. Moreover, EGFR activation induces RAS phosphorylation and subsequent activation, thereby initiating a downstream RAF→MEK→ERK signaling cascade that promotes tumor cell proliferation and anti-apoptotic signaling [31–35].

The RAS/RAF/MEK/ERK signaling axis plays pivotal roles in cell growth, proliferation, and oncogenesis [36–38]. The RAS protein, possessing GTPase activity, is activated by upstream receptors such as receptor tyrosine kinases (RTKs). Activated RAS binds to the N-terminal domain of RAF, leading to RAF activation. Activated RAF subsequently interacts with and activates downstream MEK proteins. Activated MEK further phosphorylates its sole substrate ERK. Finally, activated ERK translocates into the nucleus, triggering a series of physiological and biochemical responses. To elucidate the mechanistic basis of BF-induced antiproliferative and pro-apoptotic effects, we evaluated phosphorylation status of key pathway components in HCC cells and tumor tissues. IF, IHC staining, and Western blot analyses of HepG2 and HCCLM3 cells revealed dose-dependent suppression of p-EGFR, p-RAF, Pan RAS, p-MEK1/2, and p-ERK1/2 following BF treatment. Parallel experiments in subcutaneous and orthotopic HCC mouse models demonstrated comparable pathway suppression in tumor tissues. These findings establish that BF exerts its antitumor effects through dual inhibition of EGFR receptor activation and subsequent attenuation of RAS/RAF/MEK/ERK cascade signaling, thereby disrupting oncogenic proliferation and survival signaling in malignant hepatocytes.

Biochemically, BF’s inhibition of the RAS/RAF/MEK/ERK cascade occurred at multiple regulatory nodes, as evidenced by: [1] suppression of pathway phosphorylation events in tumor tissues [2], reduced cytoplasmic/nuclear p-ERK1/2 translocation, and [3] attenuated RAS/RAF/MEK/ERK signaling output in EGFR-overexpressing cells. This multi-level pathway modulation distinguishes BF from monotherapy agents, potentially explaining its clinical efficacy in heterogeneous HCC populations. Study limitations include the retrospective nature of clinical data collection and lack of pharmacokinetic correlation in animal models. Future investigations should incorporate: [1] multi-center prospective trials with biomarker-driven patient selection [2], combination therapy studies with immune checkpoint inhibitors, and [3] liquid biopsy-based pharmacodynamic monitoring to optimize dosing regimens.

## Conclusions

Our work establishes BF as a promising therapeutic candidate for EGFR-driven HCC, acting through dual inhibition of receptor tyrosine kinase activation and downstream RAS/RAF/MEK/ERK signaling. The convergence of clinical survival trends, pathway-specific biochemical effects, and orthogonal in vivo validation positions BF as a potential precision oncology agent warranting further clinical development.

## Supplementary Information

Below is the link to the electronic supplementary material.

Supplementary Material 1

Supplementary Material 2

## Data Availability

No datasets were generated or analysed during the current study.
