# Supplementary figures and images for: Bufalin inhibits hepatocellular carcinoma progression by blocking EGFR-mediated RAS-RAF-MEK-ERK pathway activation
Source: J Exp Clin Cancer Res. 2025 Aug 29;44:260. doi: 10.1186/s13046-025-03531-3 (PMC12395921; doi:10.1186/s13046-025-03531-3)

Figure 5A

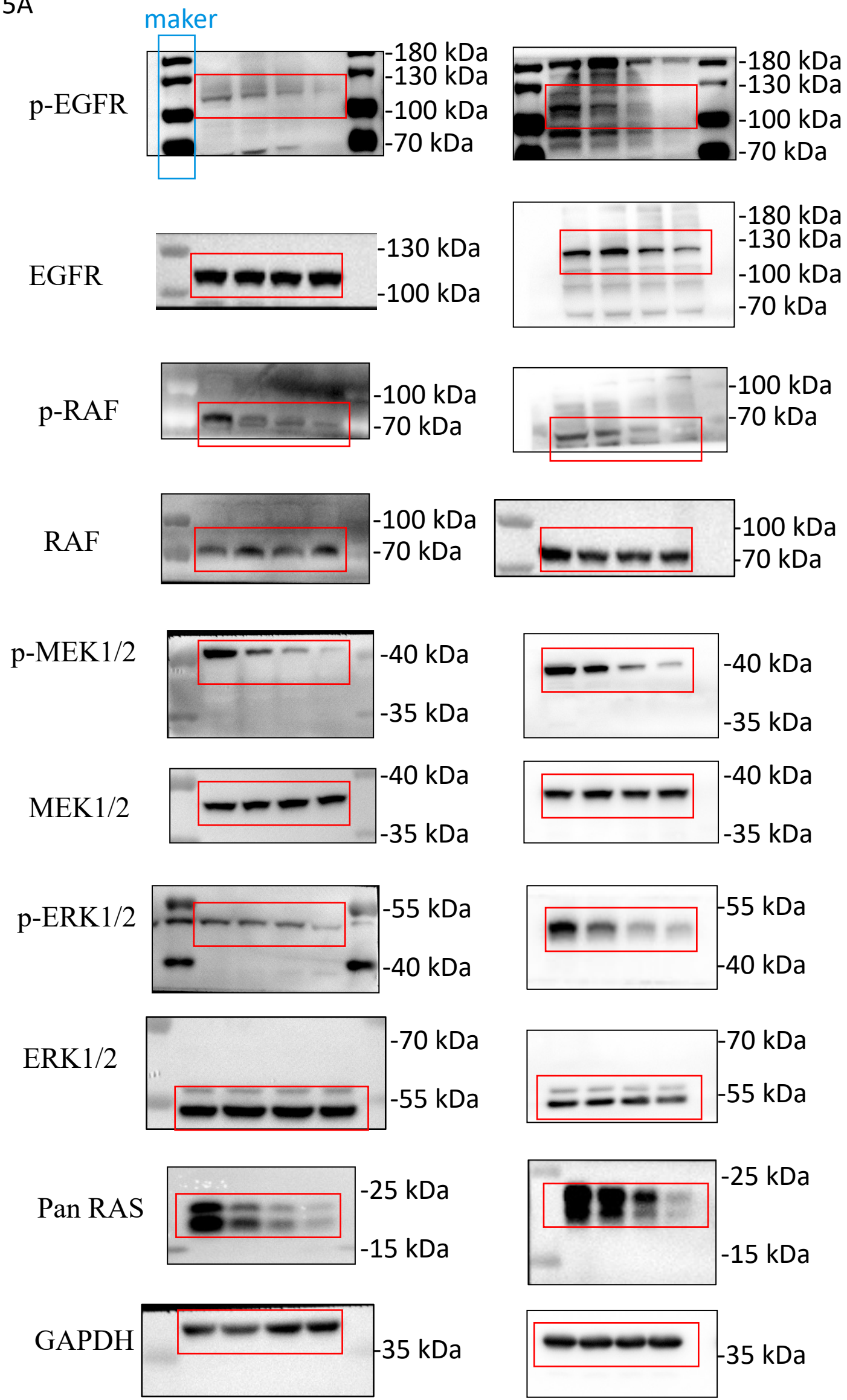

Figure 6A

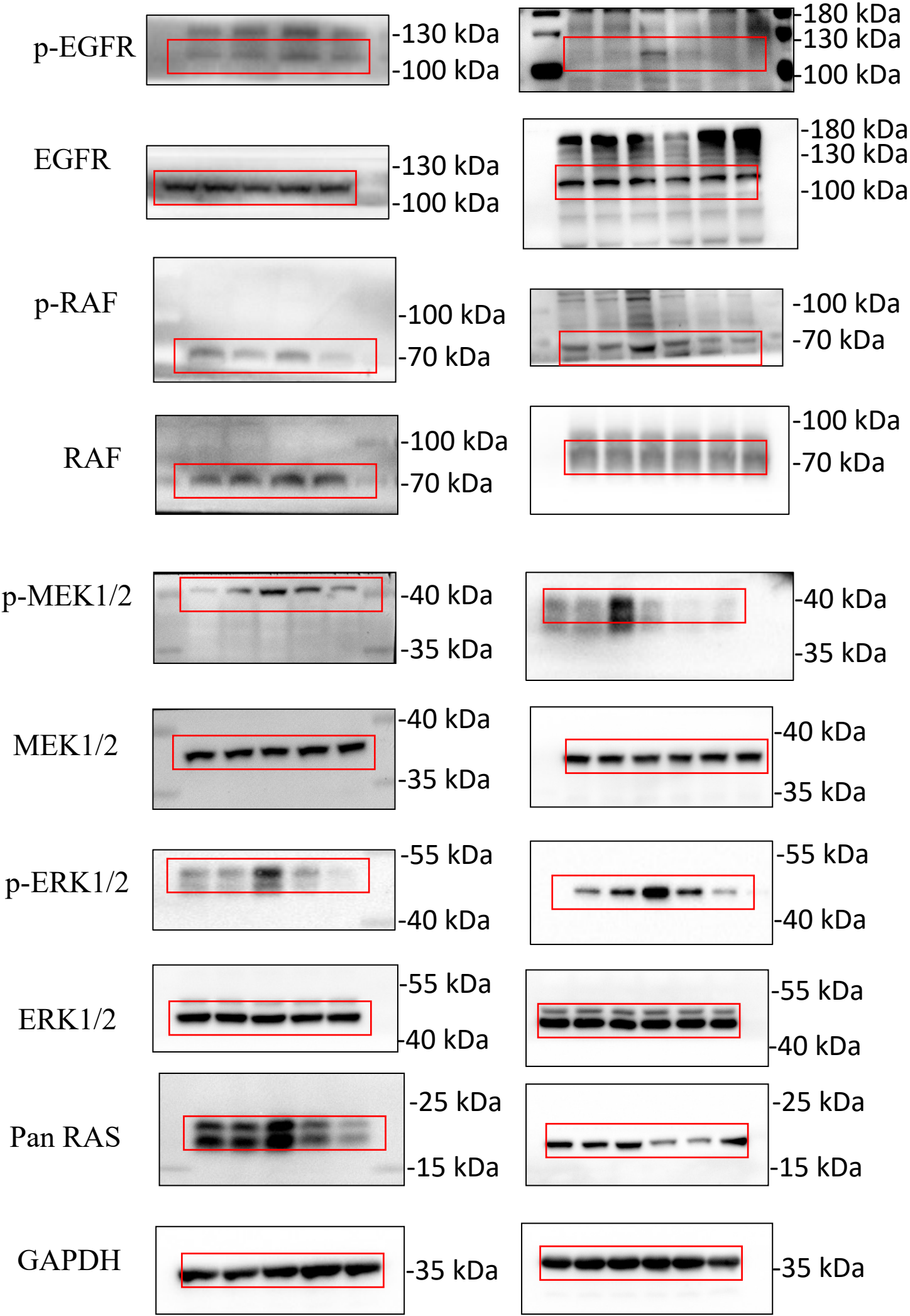

Figure 8A

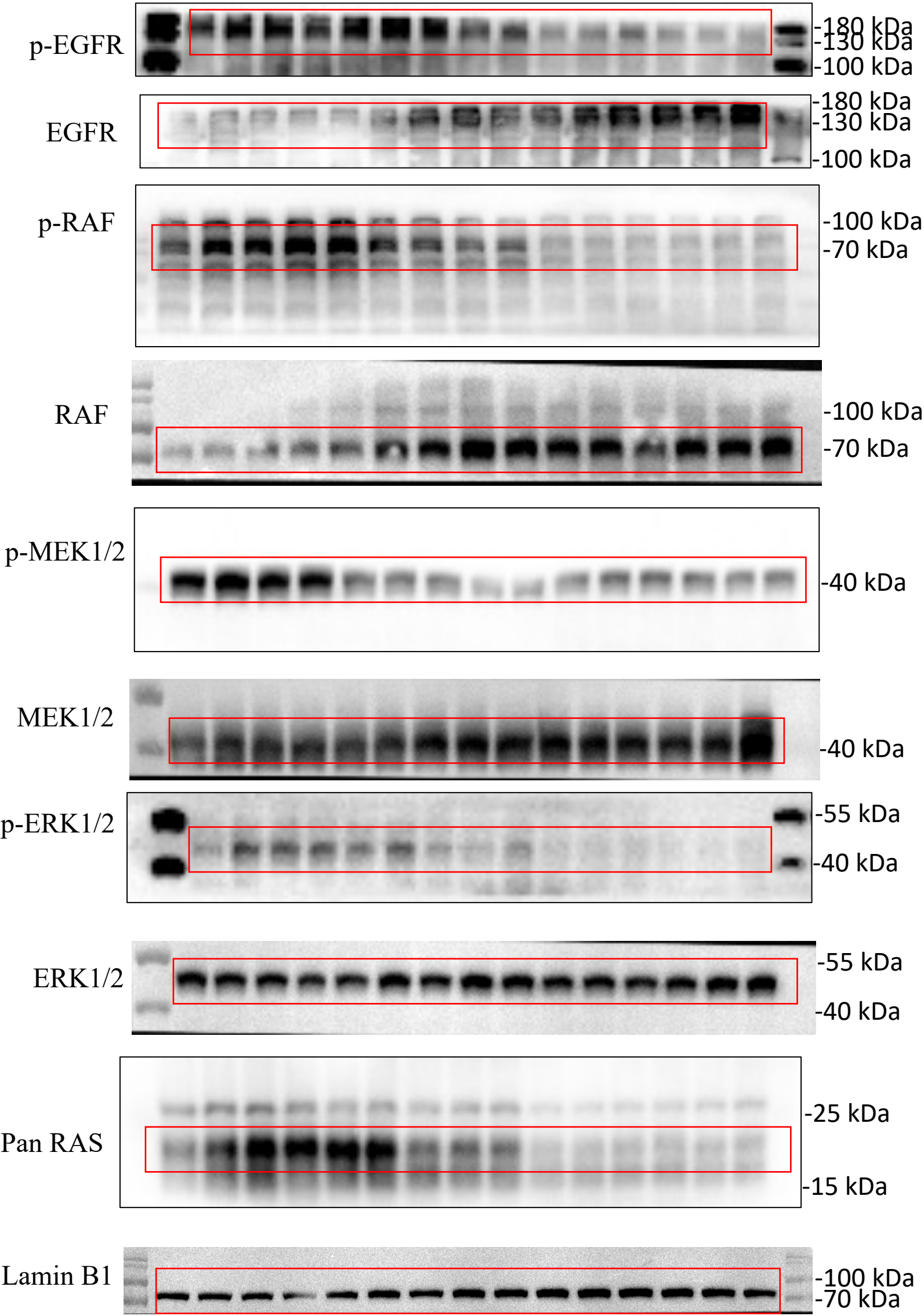

Figure 9C

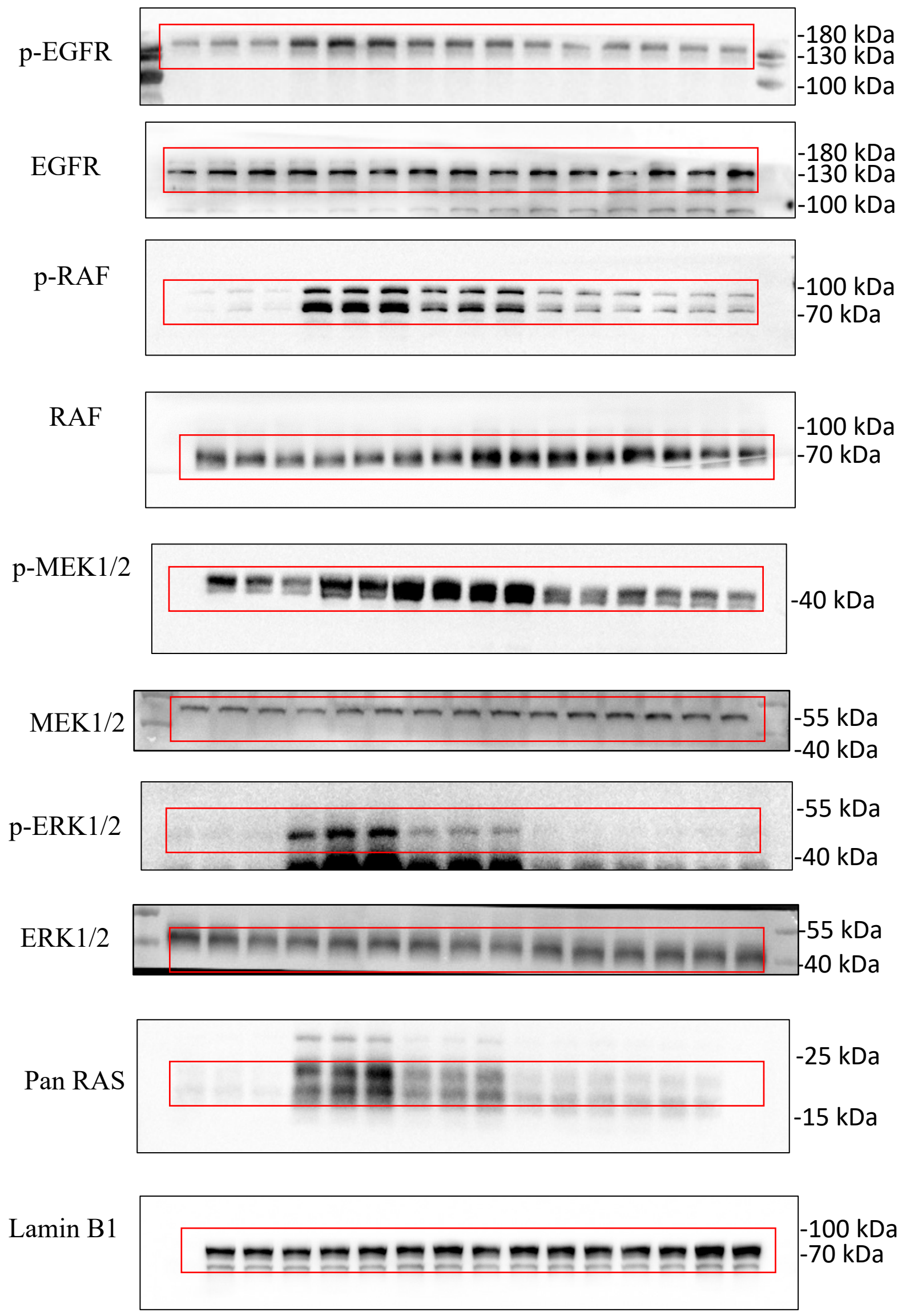

Supplement: Supplementary file 1 — Supplementary Material 1 [file 13046_2025_3531_MOESM1_ESM.pdf]
