# Supplementary material for: Bufalin inhibits hepatocellular carcinoma progression by blocking EGFR-mediated RAS-RAF-MEK-ERK pathway activation
Source: J Exp Clin Cancer Res. 2025 Aug 29;44:260. doi: 10.1186/s13046-025-03531-3 (PMC12395921; doi:10.1186/s13046-025-03531-3)
Supplement: Supplementary file 2 — Supplementary Material 2 [file 13046_2025_3531_MOESM2_ESM.docx]

**Supplementary Table 1. Demographic parameters, biochemical markers, and disease staging in two study groups**

| Characteristics | WM therapy | WM + Cinobufacini therapy | Statistics | *P* value |
| --- | --- | --- | --- | --- |
|  | (n = 53) | (n = 151) |  |  |
| Age (year) | 61.11 ± 13.41 | 60.19 ± 12.18 | 0.46(t) | 0.643 |
| Sex |  |  |  |  |
| Female | 16(30.19) | 32(21.19) | 1.76(Chi-square) | 0.184 |
| Male | 37(69.81) | 119(78.81) |  |  |
| Child-Pugh stage |  |  |  |  |
| A | 17(32.08) | 26(17.22) | 5.92(Chi-square) | 0.052 |
| B | 21(39.62) | 83(54.97) |  |  |
| C | 15(28.30) | 42(27.81) |  |  |
| BCLC stage |  |  |  |  |
| A | 0(0.00) | 1(0.66) | 0.00(Fisher) | 0.058 |
| B | 26(49.06) | 46(30.46) |  |  |
| C | 15(28.30) | 68(45.03) |  |  |
| D | 12(22.64) | 36(23.84) |  |  |
| ALB (g/L) | 27.90 ± 10.15 | 33.02 ± 8.59 | -3.56(t) | < 0.001 |
| TBIL (μmol/L) | 40.3(18.7,166.7) | 23.5(13.3,43.0) | 3.38(Z) | < 0.001 |
| PALB (g/L) | 91.3(42.8,134.5) | 101.9(54.4,142.2) | -0.68(Z) | 0.495 |
| MALB (mg/24h) | 0.2(0.2,0.2) | 0.2(0.2,0.2) | 1.37(Z) | 0.172 |
| PT (s) | 18.20 ± 9.41 | 15.12 ± 3.56 | 2.31(t) | 0.025 |
| INR | 1.36 ± 0.68 | 1.15 ± 0.27 | 2.12(t) | 0.038 |
| PTA (%) | 79.15 ± 24.09 | 83.96 ± 23.37 | -1.27(t) | 0.206 |
| AST (U/L) | 101.0(71.0,211.0) | 63.0(34.0,118.0) | 3.12(Z) | 0.002 |
| ALT (U/L) | 47.0(22.0,97.0) | 36.0(20.0,82.0) | 1.21(Z) | 0.228 |
| AFP (ng/mL) | 63.4(5.1,825.2) | 33.1(5.6,1210.0) | -0.29(Z) | 0.771 |
| CA19-9 (U/mL) | 81.2(19.2,401.4) | 31.1(13.2,82.3) | 2.46(Z) | 0.014 |
| CEA (ng/mL) | 3.0(1.9,5.7) | 3.2(2.1,5.6) | -0.03(Z) | 0.975 |
| 1 year's progress |  |  |  |  |
| No progress | 17(32.08) | 41(27.15) | 0.47(Chi-square) | 0.494 |
| Progress | 36(67.92) | 110(72.85) |  |  |
| 3 year's progress |  |  |  |  |
| No progress | 3(5.66) | 5(3.31) | 0.22(Fisher) | 0.431 |
| Progress | 50(94.34) | 146(96.69) |  |  |
| 5 year's progress |  |  |  |  |
| No progress | 0(0.00) | 4(2.65) | 0.30(Fisher) | 0.575 |
| Progress | 53(100.0) | 147(97.35) |  |  |
| 1 year's end |  |  |  |  |
| Survival | 30(56.60) | 83(54.97) | 0.04(Chi-square) | 0.837 |
| Death | 23(43.40) | 68(45.03) |  |  |
| 3 year's end |  |  |  |  |
| Survival | 7(13.21) | 24(15.89) | 0.22(Chi-square) | 0.639 |
| Death | 46(86.79) | 127(84.11) |  |  |
| 5 year's end |  |  |  |  |
| Survival | 2(3.77) | 7(4.64) | 0.30(Fisher) | 1.000 |
| Death | 51(96.23) | 144(95.36) |  |  |

Note: Normal reference ranges: ALB 35–55 g/L; TBIL 0–21 μmol/L; PALB 20–40 g/L; MALB 0–30 mg/24h; PT 9.6–13 s; INR 0.8–1.2; PTA 70–130%; AST 15–40 U/L; ALT 9–50 U/L; AFP < 25 ng/mL; CA19-9 < 37 U/mL; CEA < 5 ng/mL.


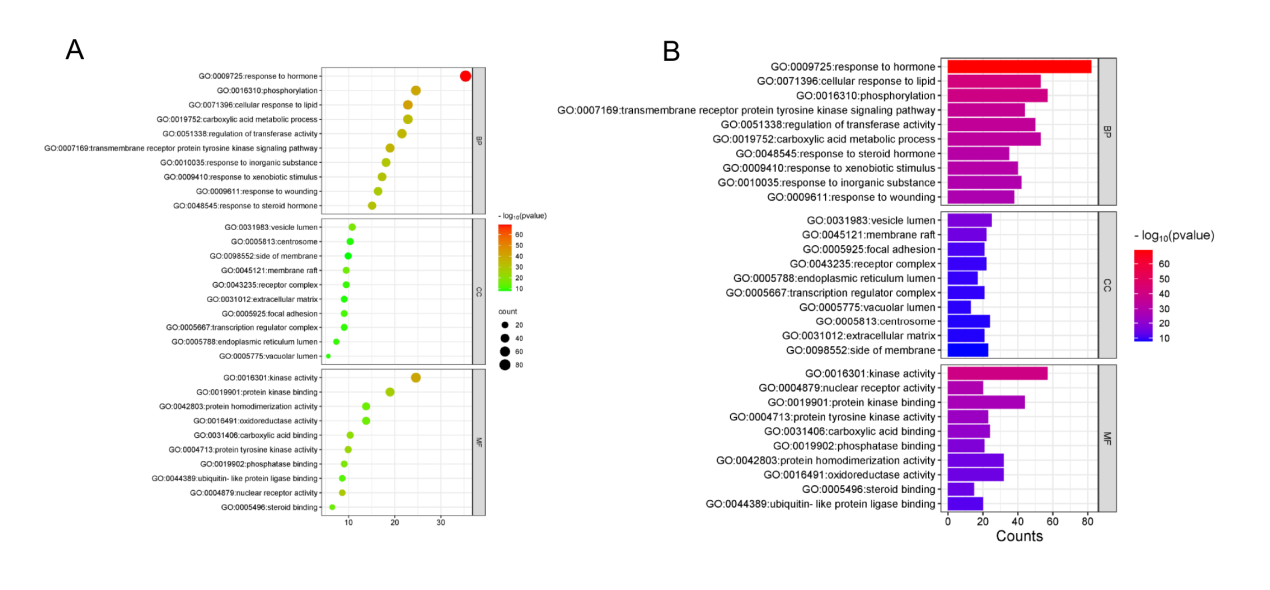


**Supplementary Fig. 1. GO enrichment analysis of key target.** **(A, B)** GO function enrichment bubble chart (**A**) and bar chart (**B**).

**
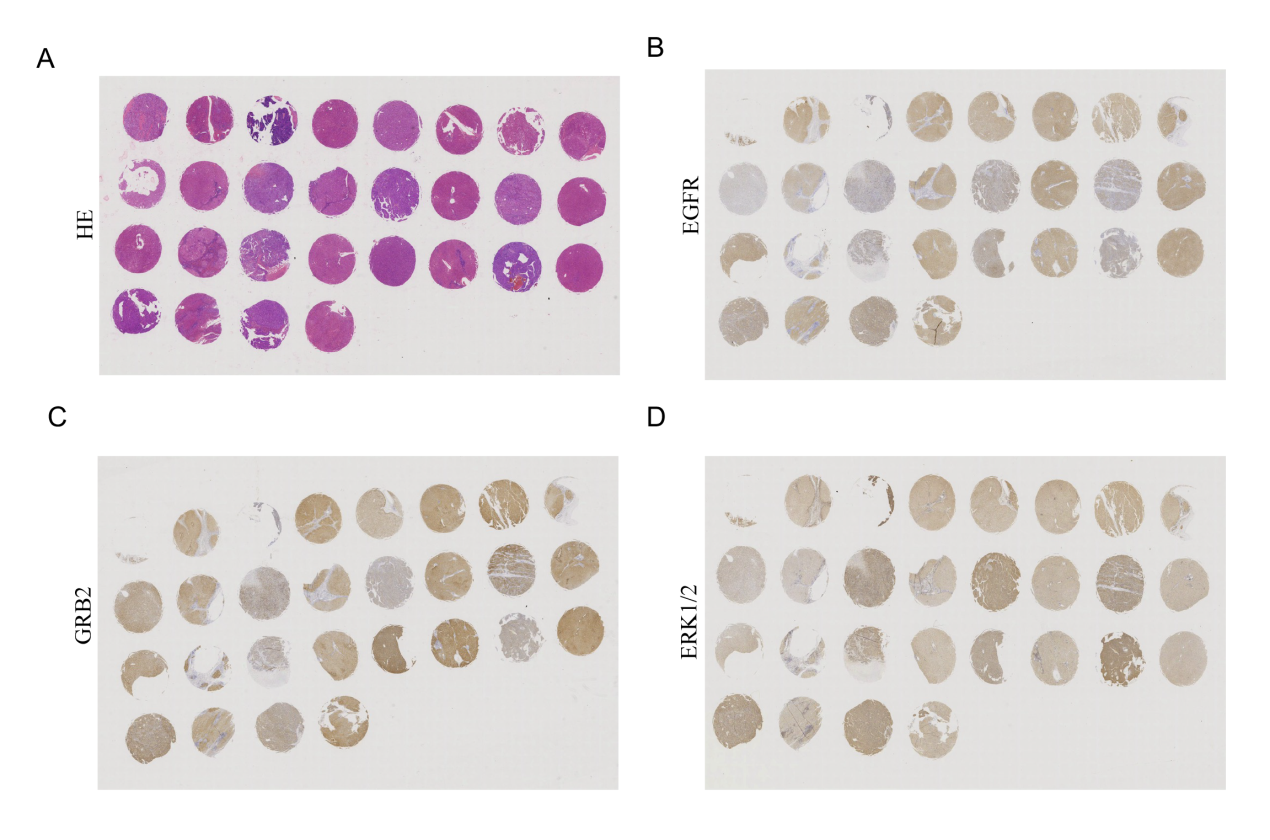
**

**Supplementary Fig. 2. HE staining, and EGFR/GRB2/ERK1/2 expression in HCC tissue microarray.** **(A)** Pathological changes of ANT tissues and HCC tissues. (B) EGFR expression in tissue microarray. (C) GRB2 expression in tissue microarray. (D) ERK1/2 expression in tissue microarray.


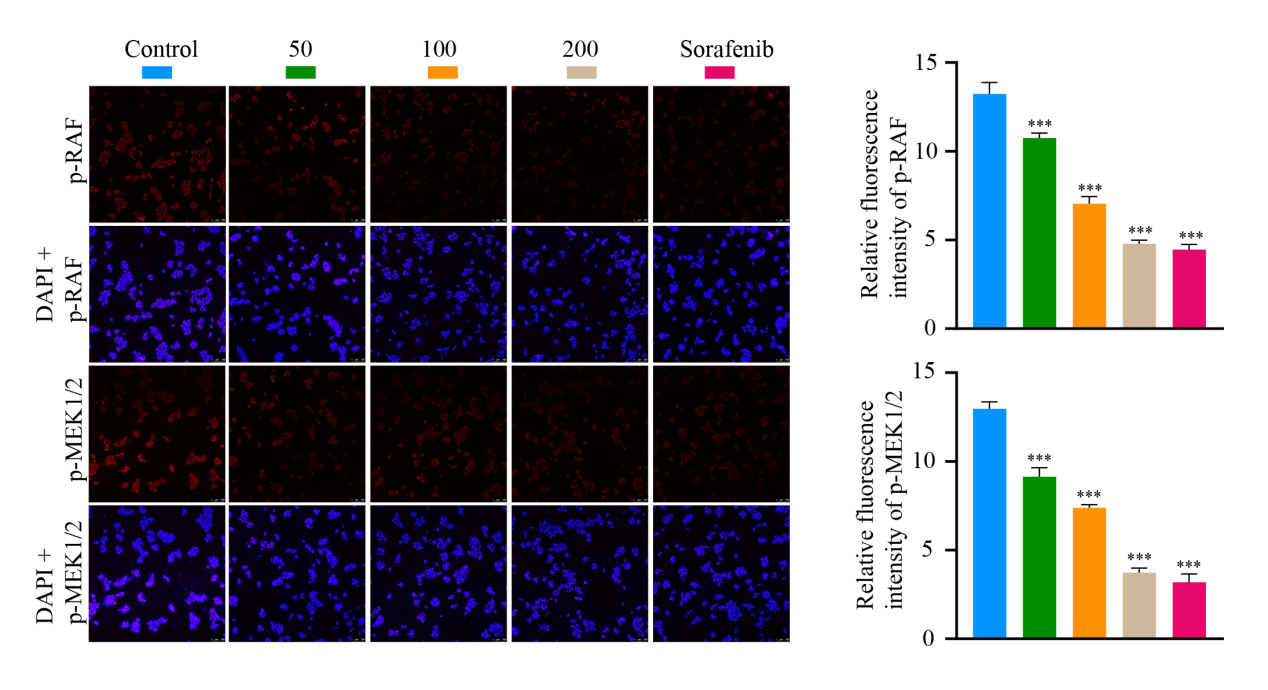


**Supplementary Fig. 3. IF staining of p-RAF and p-MEK1/2 in HepG2.** Representative pictures and relative fluorescence intensity of p-RAF and p-MEK1/2. Data are expressed as mean ± SD from three independent experiments with biological duplicates. ^***^P < 0.001 compared to the Control group.


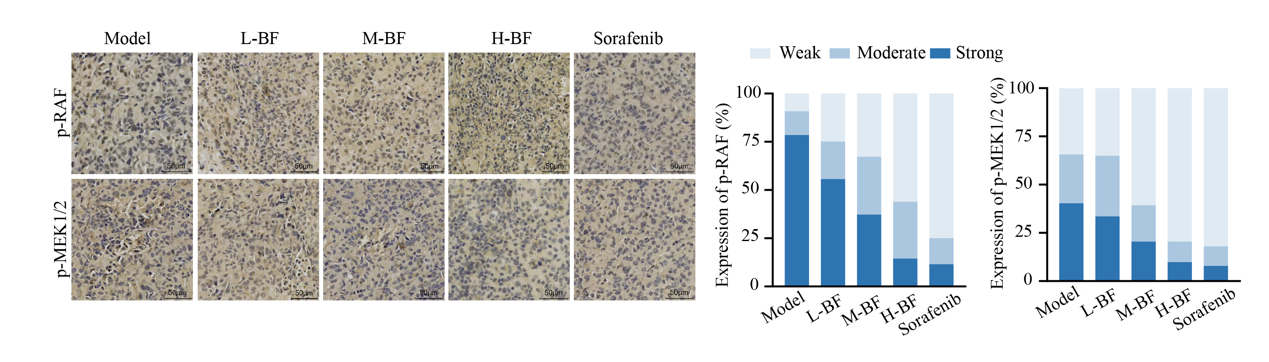


**Supplementary Fig. 4. IHC staining of p-RAF and p-MEK1/2 in subcutaneous HCC tumors.** Representative pictures and relative intensity of p-RAF and p-MEK1/2.

**
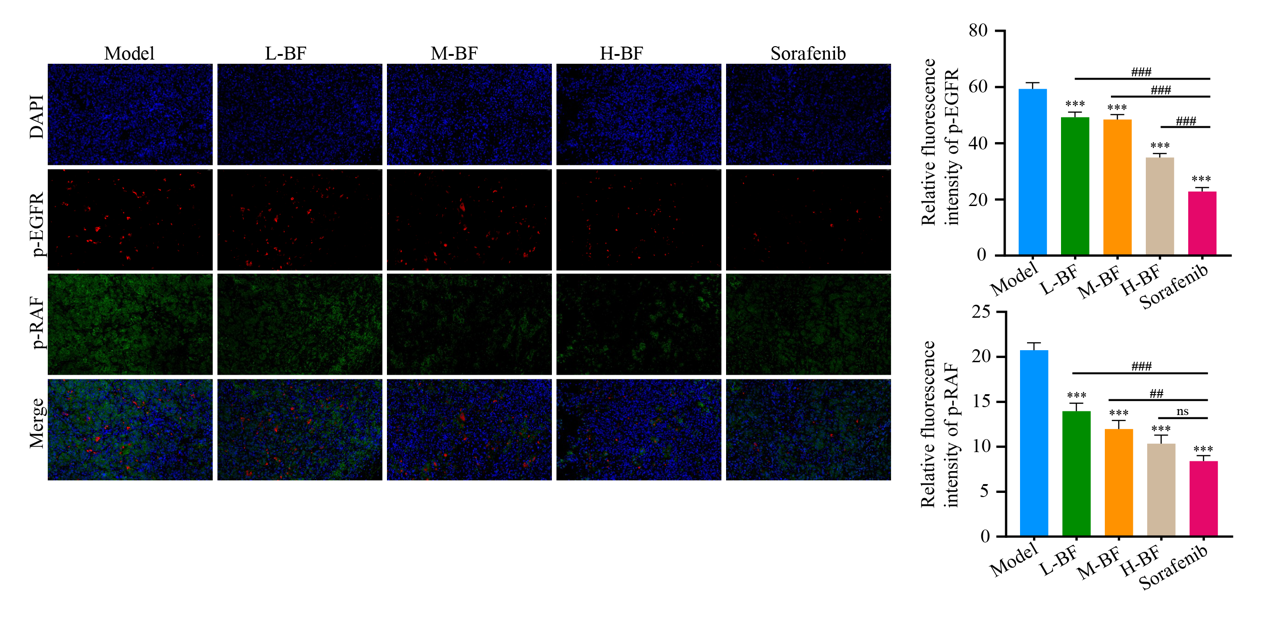
**

**Supplementary Fig. 5. IF staining of p-EGFR and p-RAF in subcutaneous HCC tumors.** Representative pictures and relative fluorescence intensity of p-EGFR and p-RAF.
